# Health and socio-demographic background of Ukrainian minors and their families in Germany - challenges for refugee medicine: A cross-sectional study from the German Network University Medicine (NUM)

**DOI:** 10.1007/s00431-024-05847-2

**Published:** 2024-12-05

**Authors:** Anna Teresa Hoffmann, Robin Kobbe, Robin Denz, Christoph Maier, Nicole Toepfner, Nina Timmesfeld, Astrid Petersmann, Katharina Giesbrecht, Olga Hovardovska, Jörg Janne Vehreschild, Margarete Scherer, Susana M. Nunes de Miranda, Lazar Mitrov, Anette Friedrichs, Amke Caliebe, Katarzyna Emilia Skipiol, Sarah Holzwarth, Yadwinder Kaur, Anne Schlegtendal, Leonhard Hojenski, Anne-Kathrin Ruß, NU(M)KRAINE Paed Study Group, Alena Akinina, Alexandra Dopfer-Jablonka, Caroline Wauschkuhn, Inna Liashenko, Maher Almahfoud, Claudia Maria Denkinger, Thomas Lücke, Jakob Höppner, Gunnar Brandhorst, Axel Hamprecht, Dmitry Sergeev, Maria J. G. T. Vehreschild, Folke Brinkmann

**Affiliations:** 1 University Children’s Hospital, Katholisches Klinikum Bochum, Ruhr-University Bochum, 44791 Bochum, Germany; 2 Institute for Infection Research and Vaccine Development (IIRVD), University Medical Centre Hamburg-Eppendorf, Hamburg, Germany; 3 Department of Infectious Disease Epidemiology, Bernhard Nocht Institute for Tropical Medicine, Hamburg, Germany; 4 Department of Medical Informatics, Biometry and Epidemiology, Ruhr University, Bochum, Germany; 5 Department of Paediatrics, Faculty of Medicine and University Hospital Carl Gustav Carus, Technische Universität Dresden, Dresden, Germany; 6 Institute of Clinical Chemistry and Laboratory Medicine, University Medicine of Oldenburg, Oldenburg, Germany; 7 Institute of Clinical Chemistry and Laboratory Medicine, University Medicine of Greifswald, Greifswald, Germany; 8 Department II of Internal Medicine, Infectious Diseases, Goethe University Frankfurt, University Hospital Frankfurt, Frankfurt Am Main, Germany; 9 Department of Epidemiology, Helmholtz Centre for Infections Research, Brunswick, Germany; 10 German Centre for Infection Research, TI BBD, Brunswick, Germany; 11 Goethe University Frankfurt, Faculty of Medicine, Institute for Digital Medicine and Clinical Data Science, Goethe University, Frankfurt, Germany; 12 Faculty of Medicine and University Hospital Cologne, Department I for Internal Medicine, University of Cologne, Cologne, Germany; 13 German Centre for Infection Research (DZIF), Partner Site Bonn-Cologne, Cologne, Germany; 14 Faculty of Medicine and University Hospital Cologne, Department I of Internal Medicine, Centre for Integrated Oncology Aachen Bonn Cologne Duesseldorf, University of Cologne, Cologne, Germany; 15 Department of Internal Medicine I, Infectious Diseases, University Hospital Schleswig-Holstein, Campus Kiel, Kiel, Germany; 16 Institute of Medical Informatics and Statistics, Kiel University and University Hospital Schleswig-Holstein, Kiel, Germany; 17 Department of Infectious Disease and Tropical Medicine, University Hospital Heidelberg, Heidelberg, Germany; 18 Section of Paediatric Pneumology, Department of Paediatrics, University of Lübeck, Campus Lübeck, Lübeck, Germany; 19 Airway Research Centre North (ARCN), Germany, Member of the German Centre for Lung Research (DZL), Grosshansdorf, Germany

**Keywords:** Ukraine, Minor refugees, Vaccination status, Infectious diseases, Intrafamilial spread, Health status

## Abstract

In 2022, over 1,000,000 people from Ukraine fled to Germany, mostly women and children. The aim of this study was to determine health status and socio-demographic background of Ukrainian refugee minors in Germany and to compare that to German minors. In this study, Ukrainian refugees of all ages, who voluntarily made contact with the study centres via flyers or refugee shelters, were interviewed with the help of trained interpreters from 09–12/2022. We assessed data from minors and their families on socio-economic status, general health perception, current and pre-existing infectious and non-contagious diseases, and vaccination status. The data for the minors were collected by interviewing the accompanying adults. In addition, a physical examination and serological screening for infectious and vaccine-preventable diseases were performed. Overall, 392 minors and accompanying adults (358) were included. Minors’ general and mental health were reported as “at least good" in 83% (316) and 80% (304), respectively. In 16% (63), infectious or other underlying diseases were recorded. Tuberculosis infections (7, 1.8%) and hepatitis C (2, 0.5%) were found through the serological tests. HIV could only be detected in one previously known case. Self-reported vaccinations against measles and varicella, for example, did not match serological findings (protection against measles 304; 84%; varicella 230; 64%).

*Conclusion*: Accompanying adults often rate the general health and well-being of Ukrainian refugee minors as rather poor. Frequently, self-reported vaccinations and preexisting diseases did not match serology results; therefore, Ukrainian refugee minors without formal vaccination certificate should be offered all recommended vaccinations and be generously screened for hepatitis B, C and HIV in addition to tuberculosis.

**Table:** 

**What is Known:** • *Infectious diseases (TB, hepatitis B/C, HIV) are more common among Ukrainian minors and vaccine hesitancy is more widespread than in many host countries and require appropriate medical care.* • *Displacement and war worsen medical care and have negative impact on mental health.*.
**What is New:** • *Although intrafamilial spread of infectious diseases was rare (in this cohort), perceived general health of Ukrainian minors is rather poor.* • *Semi-structured interviews—even with interpreters—are often insufficient to collect valid medical information.*

**Supplementary Information:**

The online version contains supplementary material available at 10.1007/s00431-024-05847-2.

## Introduction

Since the beginning of the Russian invasion of Ukraine, 1,650,000 Ukrainian refugees have been registered in Germany (as of 12.03.2024) [1]. Germany is currently the European country with the most registered Ukrainian refugees, ahead of Poland and the Czech Republic [2]. In 2022, during our study period, 80% of Ukrainian refugees were female and 80% of respondents were accompanied by minors (< 18 years of age) [1]. Minors are a particularly vulnerable group and more likely to suffer from infections as well as mental illnesses such as post-traumatic stress disorders (PTSD) [3]. In addition to this, the SARS-CoV-2 pandemic had a negative effect on the mental health of all minors, in Ukraine as well as in Germany and worldwide [4, 5].

Chronic diseases such as bronchial asthma, epilepsy and diabetes mellitus are just as common among minors in Ukraine as in Western European countries [6, 7], although the actual prevalence of chronic diseases is still difficult to estimate due to the backlogs in Ukrainian reporting systems [8]. The incidence of most infections, including vaccine-preventable diseases such as measles, is in contrast significantly higher in Ukraine than in other Western European countries, like Germany [6]. A survey conducted in the field of primary health care in Poland showed that minor refugees from Ukraine sought medical help primarily for infectious diseases (96%). Many also suffered from respiratory (27.4%) or gastrointestinal diseases (12.8%) [9].

Ukraine, with 17.7 cases per 100,000 adolescents [10] and 7.0 cases per 100,000 children under 15 years of age [11], is considered a tuberculosis (TB) high priority country with a very high prevalence of drug resistance and the highest incidence rates in the south-east of Ukraine [12].

Additionally, the number of HIV infections in Ukraine has been increasing rapidly since the 1990s with approximately 2700 HIV-infected minors (< 15 years) according to the latest UNAIDS report [13, 14]. Interestingly, more than 98% of these minors received antiretroviral therapy in 2021, while the overall treatment rate for all HIV-positive people was only 62% on average [13, 14]. The prevalences of hepatitis B and C are as well considerably higher in Ukraine than in Germany (1.3% vs 0.4% and 3.6% vs 0.3%, respectively) [15–17], and serological screening for infectious diseases in minor refugees has been recommended in Germany [18].

Vaccination rates have been low in Ukraine for many years despite national vaccination recommendations. After a large measles outbreak accompanied with high mortality rate in 2017/2018, vaccination readiness increased [6], but is still way below the > 95% target recommended by WHO (1st Measles -Mumps-Rubella (MMR) vaccination 42% in 2016, 85% in 2019) [19].

The aim of this study was to determine health status and socio-demographic background of Ukrainian refugees with a special focus on the vulnerable paediatric population and to compare results to German minors, including immunological responses to infectious and vaccine-preventable diseases. This may help to develop evidence-based recommendations for health care prevention and treatment of Ukrainian minors in Europe.

## Material and methods

### Study design

From 05.09.2022 to 21.12.2022, refugees from Ukraine were recruited as part of the cross-sectional NU(M)KRAINE study [20]. The Ukrainian participants were informed about the study via social media, family doctors and in refugee shelters to include the largest possible number of participants during the relatively short study period. Anyone who had fled Ukraine since the Russian invasion was eligible. There were no exclusion criteria. Due to this recruitment process, a response rate cannot be given. Furthermore, a formal sample size calculation has not been performed for this quantitative cross-sectional survey. Participants were divided by age into a paediatric (< 18 years, “minors”) and adult cohort (≥ 18 years). Participants were grouped by their family relationship or by a common refugee situation with the help of a shared study number.

For identified families or groups, some of the questions about the family and refugee situation were only answered once by the “head” of the respective group. The data for the minors were collected by interviewing the accompanying adults. In order to do justice to the special medical care of the highly vulnerable group of minors, we have decided to look at them again separately—together with the accompanying adults.

Semi-structural interviews (Supplement 1) were conducted with trained interpreters and a physical examination and a non-mandatory blood test were performed (see Table 1). All data were recorded in a password protected central databank (SECUtrial, electronic Case Report Form (eCRF)). All laboratory samples were analysed in the University Institute for Clinical Chemistry and Laboratory Medicine Oldenburg and data sets merged after completion of recruitment as described previously. Partially, data has been published previously [20].

**Table 1:** Overview of the serological parameters measured. Assays were performed according to the manufacturer’s recommendations, further details see [20]

Assay/reagent name	Result type	Manufacturer	Instrument
Anti-**Diphtheria**-Toxoid-ELISA IgG	Quantitative	Euroimmun, Luebeck, Germany	Analyzer I
Anti-**Tetanus**-Toxoid-ELISA IgG	Quantitative	Euroimmun, Luebeck, Germany	Analyzer I
Elecsys **Anti-SARS-CoV-2**	Qualitative	Roche Diagnostics Germany GmbH, Mannheim, Germany	Cobas e 801 Analysis module
Elecsys **Anti-SARS-CoV-2 S**	Quantitative	Roche Diagnostics Germany GmbH, Mannheim, Germany	Cobas e 801 Analysis module
Elecsys **Rubella** IgG	Quantitative	Roche Diagnostics Germany GmbH, Mannheim, Germany	Cobas e 801 Analysis module
Elecsys **Anti-HAV**	Qualitative	Roche Diagnostics Germany GmbH, Mannheim, Germany	Cobas e 801 Analysis module
Elecsys **HIV** Duo	Qualitative	Roche Diagnostics Germany GmbH, Mannheim, Germany	Cobas e 801 Analysis module
Elecsys **HBs-Ag**	Qualitative	Roche Diagnostics Germany GmbH, Mannheim, Germany	Cobas e 801 Analysis module
Elecsys **Anti-HBc**	Qualitative	Roche Diagnostics Germany GmbH, Mannheim, Germany	Cobas e 801 Analysis module
Elecsys **Anti-HBs**	Quantitative	Roche Diagnostics Germany GmbH, Mannheim, Germany	Cobas e 801 Analysis module
Elecsys **Anti-HCV**	Qualitative	Roche Diagnostics Germany GmbH, Mannheim, Germany	Cobas e 801 Analysis module
**Haemophilus influenzae B** IgG ELISA	Quantitative	DRG Instruments GmbH, Marburg, Germany	Microplate-Reader, Tecan
Human Anti-**Polio** Virus 1–3 IgG ELISA	Quantitative	ALPHA DIAGNOSTIC INTERNATIONAL, San Antonio, USA	Microplate-Reader, Tecan
LIAISON® **Bordetella pertussis** Toxin IgG	Quantitative	DiaSorin S.p.A., Dietzenbach, Germany	LIAISON® XL
LIAISON® **VZV** IgG	Quantitative	DiaSorin S.p.A., Dietzenbach, Germany	LIAISON® XL
LIAISON® **Mumps** IgG	Quantitative	DiaSorin S.p.A., Dietzenbach, Germany	LIAISON® XL
LIAISON® **Measles** IgG	Quantitative	DiaSorin S.p.A., Dietzenbach, Germany	LIAISON® XL
**QuantiFERON**®-**TB** Gold Plus LIAISON® Kit, QuantiFERON® Blood Collection Tubes	Qualitative	DiaSorin S.p.A., Dietzenbach, Germany QIAGEN GmbH, Hilden, Germany	LIAISON® XL
**QuantiFERON®-SARS-COV-2** LIAISON® Kit, QuantiFERON® Extended Set Blood Collection Tubes	Qualitative	DiaSorin S.p.A., Dietzenbach, Germany QIAGEN GmbH, Hilden, Germany	LIAISON® XL

Importantly, all TB-IGRA positive participants or those with other findings requiring treatment were informed and guided to further investigations and treatment.

Descriptive statistical evaluation and additional analyses using the chi-square test and Pearson correlation were performed with the statistics software SPSS (Version 29). 95% confidence intervals (*z* = 1.96) were calculated for sample sizes *n* > 5 with

$$p\pm z\times \sqrt{\frac{p\times (1-p)}{n}}$$

### Ethics

Two different leading ethics committees evaluated the study protocol, the one of the Goethe University in Frankfurt for adult participants (nr. 2022–831) and of Ruhr University in Bochum for the minor cohort (nr. 22–7623). Subsequently, the study was approved by all local ethics committees of all study centres.

## Results

### Subjects

Based on the inclusion criteria, 750 (42%) out of a total of 1793 participants of the NU(M)KRAINE study were included, 358 adults and 392 minors. 214 (55%) of the minors were female, about half (205; 52%) were ≤ 12 years. The participants belong to 275 “groups”, further referred to as “families”. 2.5% (10/392) were single unaccompanied minors. The majority (222/275; 80%) of the families indicated a family size per household of two to four persons, whereby they often were not able to travel together as complete families. Only 13% (45) of the adults were fathers, whereas 70% (250) were mothers (Table 2). Slightly more than half of the families (157) came from larger cities (≥ 500,000 inhabitants), especially from Kiev and Eastern Ukraine. Most participants had higher education with 86% of the mothers (215/250) holding at least a secondary school diploma (Table 2).

**Table 2:**
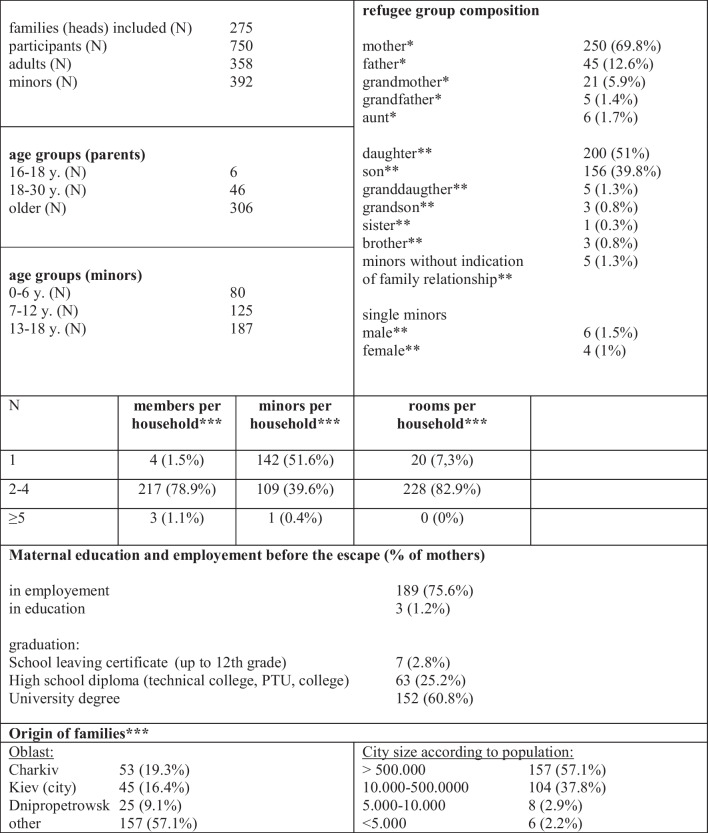
Description of the cohort

For all questions, it was possible not to give an answer. These missing answers are not always listed in the table. In this case, the difference to 100% corresponds to the missing information. *% per adults. **% per minors. ***% per head. One person was declared as the “head” for each family. Some questions about socio-demographics and the flight were only asked of this person

### Medical history and physical examination

Eighty-three of the minors who answered the question (381) and 75% of the adults (268/357) were in overall “at least good” (i.e. good, very good or excellent) general health (Table 3). Mental health of Ukrainian minors was also assessed as being rather poor (“at least good” 80% (304/380)) but none of the minors felt sad or lonely. No differences related to gender were observed. Although 3% of the Ukrainian minors reported “poor” state of health, only one of these minors needed immediate care after medical evaluation (Table 3).

**Table 3:** Health status, symptoms and need of immediate care in minors

	**general health** **status reported**	**mental health status reported**			
	**all** (% of minors [95% CI])	**all** (% of minors [95% CI])	**need of immediate care** (% of mental health status)		
excellent	47 (12% [8.5–15%])	56 (14% [11–18%])	0 (0%)		
very good	96 (25% [20–29%])	87 (22% [18–26%])	0 (0%)		
good	173 (44% [39–49%])	161 (41% [36–46%])	12 (8%)		
reasonably good	52 (13% [10–16.5%])	61 (16% [12–19%])	3 (5%)		
bad	13 (3% [1.5–5%])	15 (4% [2–5.5%])	2 (13%)		
**loneliness reported**	0 (0%)				
**sadness reported**	0 (0%)				
**Reported** **Symptoms**	**all** (% of minors [95% CI])	**need of immediate care** (% of all)	other reported symptoms or diseases*	**all** (% of minors [95% CI])	**need of immediate care** (% of all)
fever	102 (26% [21.5–31.5%])	6 (6%)	allergies	3 (0.8%)	0 (0%)
loss of appetite	84 (21% [17.5–25.5%])	9 (11%)	symptoms of upper respiratory tract infection	6 (1.5% [0.5–2.5%])	1 (17%)
swelling of lymph nodes	42 (11% [7.5–14%])	4 (10%)	malnutrition/ eating disorder	3 (0.8%)	1 (33%)
headache	128 (33% [28–37.5%])	7 (6%)	pain	4 (1%)	0 (0%)
night sweats	41 (11% [7.5–13.5%])	1 (2%)	chronic illnesses	3 (0.8%)	1 (33%)
undesirable weight loss	21 (5% [3–7.5%])	0 (0%)	panic attacks/ anxiety	1 (0.3%)	0 (0%)
cough productive (% of cough)	116 (30% [25–34%]) 53 (46% [41–50.5%])	7 (6%) 3 (6%)	fatigue/exhaustion	2 (0.5%)	0 (0%)
dyspnea	21 (5% [3–7.5%])	3 (14%)	other	7 (1.8%)	1 (14%)
gastrointestinal symptoms (e.g., diarrhoea, abdominal pain, constipation)	80 (20% [16.5–24.5%])	9 (11%)			
neurological symptoms (e.g., paralysis, gait disturbance, double vision)	19 (5% [2.5–7%])	5 (26%)			

The accompanying adults (and older adolescents themselves) were asked about physical and mental symptoms over the past three months as well as their general and mental health status. The physical examination and assessment of whether a minor was in need of acute treatment was carried out by the study doctor on site. *Data from free text entries were summarized thematically for evaluation purposes; % results > 2 have been rounded and 95% CI > 2 have been rounded in 0.5% steps for clarity. 95% CI not shown for 0% and not for “need of immediate care” analyses. 95% CI shown only for *n* > 5

Medical history of chronic or infectious diseases were reported in 16% of minors. In addition, three minors were declared as suffering from a chronic illness as their “symptoms” matched this definition of our survey. 11% of minors were underweighted (BMI < 3rd WHO/CDC percentile) and 7% obese (> 97th) (Table 4).

**Table 4:** Medical history of illness (chronic and infectious) and underweight/obesity in minors

Medical history of illness	*n* (% [95% CI])			
**Any medical history of illness**	**63 (16% [12–20%])**			
**More than one**	**19/63 (30% [19–41%])**			
Congenital anomalies*	19 (5% [3–7%])			
Developmental delay*	22 (6% [3–8%])			
Bronchial asthma*	5 (1.3%)			
Drug therapy**	2 (40%)			
Bronchitis*	20 (5% [3–7%])			
Drug therapy**	6 (30% [9–50%])			
Diabetes mellitus*	2 (0.5%)			
Insulin therapy**	2 (100%)			
Epilepsy*	5 (1.3%)			
Drug therapy**	5 (100%)			
Mental disorders*	7 (1.8% [0.5–3%])			
Drug therapy**	4 (57.1%)			
Solid tumor disease**	2 (0.5%)			
Drug therapy**	1 (50%)			
Hematooncological disease*	2 (0.5%)			
Drug therapy**	2 (100%)			
Immunodeficiency/immune disease*	5 (1.3%)			
Drug therapy**	3 (60%)			
Tuberculosis*	2 (0.5%)			
Localization**	0 (0%)			
Initial diagnosis**	0 (0%)			
TBC-specific therapy**	2 (100%)			
Is drug resistance known?**	1 (50%)			
Have drugs other than standard therapy been used?**	0 (0%)			
Has therapy been completed?**	1 (50%)			
Was TBC therapy interrupted due to/during escape?**	0 (0%)			
HIV infection*	1 (0.3%)			
Drug therapy**	0 (0%)			
**Underweight and obesity**	**All** (% [95% CI])	** < 7 y** (% of age group [95% CI])	**7–12 y** (% of age group [95% CI])	**13–18 y** (% of age group [95% CI])
< P3 WHO/CDC				
All*	43 (11% [8–14%])	19 (24% [14.5–33%])	14 (11% [5.5–16.5%])	10 (5% [2–8.5%])
Male***	19 (11% [6–15%])	6 (17% [4.5–29.5%])	6 (10% [2.5–18%])	7 (8% [2.5–14.5%])
Female****	24 (11% [7–15.5%])	13 (13% [3–22.5%])	8 (12% [4–20%])	3 (3%)
< P3 Ukrainian (according to [21])				
All*	7 (1.8% [0.5–3%])	Not available	3 (2%)	4 (2%)
Male***	4 (2%)	Not available	0 (0%)	4 (5%)
Female****	3 (1.4%)	Not available	3 (5%)	0 (0%)
> P97 WHO/CDC				
All*	27 (7% [4.5–9.5%])	11 (14% [6–21%])	10 (8% [3–13%])	6 (3% [0.5–5.5%])
Male***	14 (8% [4–12%])	4 (11%)	6 (10% [2.5–18%])	4 (5%)
Female****	13 (6%)	7 (7%)	4 (6%)	2 (1.9%)
> P97 ukrainian (according to [21])				
All*	40 (10% [7–13%])	Not available	16 (13% [7–18.5%])	24 (13% [8–17.5%])
Male***	23 (13% [8–18%])	Not available	9 (15% [6–24.5%])	14 (17% [9–25%])
Female****	17 (8% [4.5–11.5%])	Not available	7 (11% [3–18%])	10 (10% [4–15.5%])

Analysis of all anamnestic data on medical history and calculation of obesity and underweight using the WHO/CDC percentiles [22] and the Ukrainian percentiles [21] for BMI. * % all minors (*n* = 392) and by age group (< 7 y *n* = 80, 7–12 y *n* = 125, > 12 y *n* = 187). ** % in relation to the specified disease. *** % all boys (*n* = 177) and by age group (< 7 y *n* = 35, 7–12 y *n* = 59, > 12 y *n* = 83). **** % all girls (*n* = 214) and by age group (< 7 y *n* = 45, 7–12 y *n* = 66, > 12 y *n* = 103)

% results > 2 have been rounded and 95% CI > 2 have been rounded in 0.5% steps for clarity. 95% CI not shown for 0% and not for subgroup analyses. 95% CI shown only for *n* > 5

The most common symptoms in the three months prior to the interview reported were headache (33%), cough (30%), fever (26%) and gastrointestinal problems (20%). Of those minors (17; 4%) who needed immediate care after medical examination, 14 (82%) had ≥ 2 reported acute symptoms. Although complaints were common, pathological findings on physical examination were rare (< 5%).

### Immunological responses to infectious pathogens

Out of a total of 46 positive TB-Interferon-Gamma-Release Assays (TB-IGRA), 7 were detected in minors, all of them were older than 6 years. Interestingly, only in 8 cases (17%) was the TB-IGRA positive in more than one person per family. Six of the 7 minors had a latent tuberculosis infection (LTBI); one was lost to follow- up (Table 4 and Table 5).

**Table 5:** Infectious diseases—medical history and serological evidence

	Tuberculosis*	HIV	Hepatitis B	Hepatitis C	SARS-CoV2
Serological test	IGRA	HIV Ag/Ak	HBs-Ag	Anti-HCV	IGRA
**Known medical histories**					
At home**	1 (0.4%)	-	-	-	164 (60% [54–65.5%])
Adults***	-	3 (0.8%)	3 (0.8%)	4 (1.1%)	-
Minors****	-	1 (0.3%)	-		-
**Positive test result**					
Adults***	39 (11% [7.5–14%])	5 (1.4%)	4 (1.1%)	14 (3.9% [2–6%])	262 (73% [68.5–78%))
Minors****	7 (1.8% [0.5–3%])	1 (0.3%)	0 (0%)	2 (0.5%)	180 (46% [41–51%])
Tests positive within one family**					
Only 1 person affected	36 (13% [9–17%])	4 (1.5%)	4 (1.5%)	14 (5% [2.5–7.5%])	-
Another person affected	8 (3% [1–5%])	2 (0.7%)	0 (0%)	2 (0.7%)	-
**New diagnoses** (positive test, not preknown)°					
Adults	-	2 (40%)	1 (25%)	10 (71% [47.5–95%])	-
Minors	-	0 (0%)	-	-	-
**SARS CoV2 antibody testing and vaccination**					
	**Nucleocapsid (N)**	**Spike (S)**	**Both (N + S) positive**		
Total number of positive tests°°	684 (91% [89–93%])	708 (94% [92.5–96%])	683 (91% [89–93%])		
Adults***	335 (94% [91–96%])	351 (98% [96.5–99.5%])	334 (93% [90.5–96%])		
Minors****	345 (88% [85–91%])	343 (88% [84–91%])	335 (86% [82–89%])		
**Vaccination status in total°°**	277 (36.9% [33.5–40%])				
Adults***	238 (66.5% [61.5–71.5%])				
Minors****	39 (10% [7–13%])		** < 5 y**	**5–11 y**	** ≥ 12 y**
Minors by age approval of the vaccination°°°			1 (2%)	4 (3%)	34 (18% [12.5–23.5%])

Presentation of serological evidence in adults and minors in relation to family relationships and in connection with anamnestic data. *The medical history for tuberculosis in minors is listed separately in Table 4 and is not shown here.—data not available or not shown here. ** % of families (*n* = 275). *** % of adults (*n* = 358). **** % of minors (*n* = 392). °° % of all participants (*n* = 750). °°° % of age group by age approval of vaccination (< 5 y *n* = 49; 5–11 y *n* = 156; ≥ 12 y *n* = 187). % results > 2 have been rounded and 95% CI > 2 have been rounded in 0.5% steps for clarity. 95% CI not shown for 0% and not for subgroup analyses. 95% CI shown only for *n* > 5

Two cases of chronic hepatitis C in minors were identified (11 and 14 years of age) by serological screening, while the only HIV-positive 6-year-old child was known to be HIV-positive and already received antiretroviral treatment. No chronic hepatitis B infection was detected in any minor (Table 5).

All participants were asked about their vaccination status and a serological measurement of spike and nucleocapsid antibodies, as well as specific T cell responses by SARS-CoV2-IGRA were performed. Antibodies were detected in both, minors and adults in around 90% (Table 5). The percentage of T cellular responses was lower with 45.9% positive IGRAs.

### Antibody detection in vaccine-preventable diseases

**Fig. 1:**
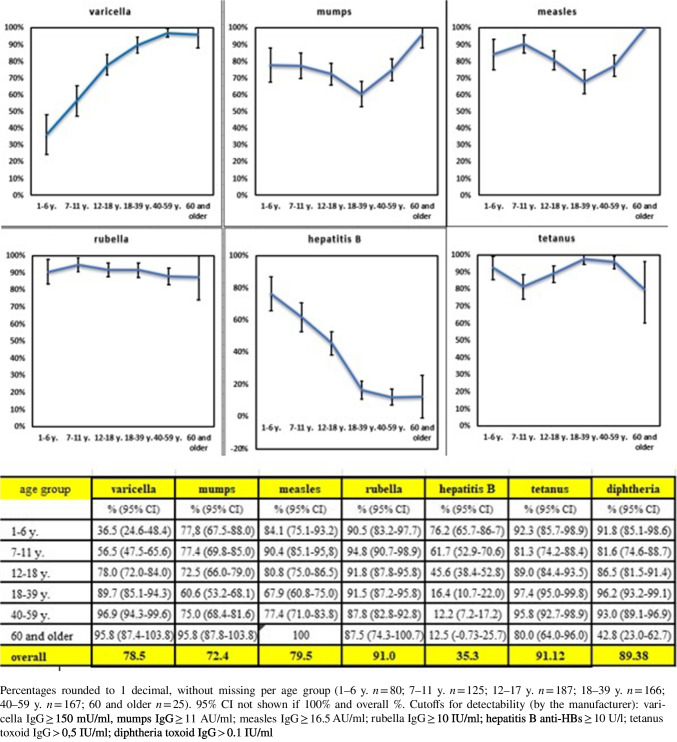
Detectability of antibodies for vaccine preventable diseases

Humoral immune response against vaccine-preventable diseases were analysed (Table 1). The prevalence of antibodies against polio (100%, all ages), tetanus, diphtheria, haemophilus influenzae (99–100%, all ages) and rubella were very high (see Fig. 1), whereas a serological correlate of protection against measles (defined as IgG ≥ 16.5 AU/ml) was detected in only 84% (304/360) of minors. Our data confirms that only a history of chicken pox reported by 52% (204) of all minors matches serological correlates of protection (IgG ≥ 150 mU/ml). A correspondingly high IgG was detectable in 88% (180/204), with the probability of a positive result increasing with age (< 7 y. 71% vs. > 12 y. 93%), as shown in Fig. 1. In contrast, the detectability of hepatitis B antibodies decreases while for measles and mumps it shows a gap in adolescents and young adults. Importantly, not all children with a serological correlate of protection also had a positive vaccination history.

### General vaccination attitude

During our interviews, two thirds of participants (adults and minors ≥ 7 years) stated that they generally consented to receiving the recommended vaccinations in Ukraine. In parents of younger children (< 7 years), this rate slightly increased to 75%. Around 15% of respondents generally were agreed with the vaccine recommendations but had reservations about individual vaccines (not specified here).

## Discussion

### Sociodemographic and health-status

Most refugee minors and their respective families came from Kiev and larger Easter Ukrainian cities and were generally well educated. These findings are consistent with a larger-scale nation-wide survey in Germany in 2022 (11,753/48,000 Ukrainians contacted by post), with 80% female participants, 72% with university degree and about a third came from Charkiv (15%) or Kiev (19%). The predominance of women in both surveys results from the fact that men were only allowed to leave Ukraine if they were fathers of more than three children or had a handicapped child [1].

During the same period, we conducted a study of minors who had grown up in Germany, in which the same questionnaire was used to assess their general and mental health. Although two different populations are never exactly comparable, the German minors showed markedly better results (“at least good” general health 93% (732/783); mental health 91% (707/777)) [21, 22]. Reasons for poorer health perception could be traumatic experiences of armed conflict, flight and uprooting [3]. Well-being has been linked to regular school or daycare attendance [1], which emphasizes the need for rapid integration. However, it remains essential to investigate psychological distress and to provide psychological diagnostics and treatment if necessary in the native language [23].

Most reported diseases were bronchitis, congenital anomalies and developmental delay (each around 5%), other chronic diseases like bronchial asthma and diabetes mellitus corresponding with 1.3% and 0.5% to the prevalence reported for Ukraine [6], while epilepsy was less frequent with only 1.3% [7]. None of these diseases was more common in German minors.

Importantly, underweight (< P3) seemed to be more common in Ukrainian minors and might represent a significant health problem (11% vs. 7.5%, *p* < 0.05 [24]) with almost a quarter of under 7-year-olds affected. Therefore, growth and weight monitoring should be given special attention in care of Ukrainian minors. Interestingly, we noticed a discrepancy between the incidence of underweight (e.g. 13–18 years 2.1% instead of 5.3%, *p* = 0.1) and obesity (e.g. 13–18 years 12.8% instead of 3.2%, *p* < 0.01) when using the WHO percentiles compared to the percentiles published for Ukraine in 2018 [25], illustrating the lack of comparability between country-specific percentiles and WHO standards [26].

### Infectious diseases and intra-family transmission

Regarding the data on SARS-CoV-2 in Ukrainian minors in comparison to the German minors from our studies mentioned above, antibodies against SARS-CoV-2 were detectable more often (spike antibodies 87.5% vs. 85.5%; nucleocapsid antibodies 88% vs. 67.2% [21]), while T cellular response were less often positive (IGRA 45.9% vs. 67.6% [27]), most likely due to a lower vaccination rate resulting in hybrid immunity.

In addition to vaccine hesitancy in Ukraine, lack of vaccine availability and the delayed implementation due to the war probably played a role. The role of cellular immune response for protection against SARS-CoV2 is still unclear, but vaccination—at least for the risk of chronically ill minors—is for Germany, for example strongly recommended [28].

Although TB incidence among Ukrainian minors is lower than in adults (1.8% vs. 10.9%) [29], it is still higher than in minors living in Germany (estimated incidence 7/100,000 vs. 1.3/100,000, [11]). With regard to the high prevalence of TB and very high level of drug-resistance in Ukraine, the low rate of positive TB IGRA in minors seems to be reassuring. However, it could also be biased by an assumable better socioeconomic status of this group. The risk to develop potentially infectious TB within the first 2 years after immigration is increased in refugees. Therefore, we recommend TB screening for all Ukrainian minor refugees in line with national regulations, even if they do not live in shared accommodation. History of contact had not been a reliable source of information in our cohort (e.g. for two children from different families, known TB was reported, but only one person reported household contact with TB).

The incidences for HIV (0.3%) and hepatitis C (0.5%) were higher than in age-matched German minors, but below expected occurrence of up to 1% HIV and 3.6% hepatitis C [15–17]. Multiple cases per family were also a rarity (HIV and hepatitis C 0.7% of cases each). Only for hepatitis C, several new diagnoses among the accompanying adults were detected, while most HIV and hepatitis B infections were already known. Screening for infectious diseases like HIV, hepatitis B and C should nevertheless be offered to all minors and their families [6, 15, 30].

### Immune protection for vaccine-preventable diseases in minors and their families

Testing serological correlates of protection against vaccine-preventable diseases combined with a survey of respective history of vaccination status were essential parts of this study. In Ukraine, vaccinations are usually only documented by the healthcare provider who administered them [30], so many of our participants did not have a vaccination record. Therefore, the accuracy of the individual immunization history becomes important. History of chickenpox is a reliable marker for serological correlate of protection against varicella, while for other vaccine-preventable diseases history of infection insufficiently correlates with protection.

While we found broad antibody prevalence for polio, tetanus, diphtheria, haemophilus influenzae and mumps, serological protection against measles, varicella and hepatitis B is much lower. In principle, all undocumented and necessary vaccinations should therefore be carried out in accordance with the recommendations of the WHO [31] or the host countries (e.g. [28]).

We would like to focus on measles and hepatitis B in detail as examples.

Measles vaccination is one of the most important international public health intervention available, with > 90% effectiveness [32]. In Germany—as example for Western Europe—vaccination rates for measles are high with 97.4% for 1st vaccination [33], whereas serological protection rate against measles in the study cohort was lower (84.4%) and far below the WHO > 95% target. In a previous European survey, 86% of mothers from nine countries stated that their children had been vaccinated [34] and a German study [33] reported that 7.7% (6.5–9.1) of parents did not have their children vaccinated for fear of side effects. In Ukraine on the other hand, vaccine hesitancy has been very high [35]. Only after a large national measles outbreak in 2018 causing notable mortality in children, measles vaccination rates did increase [6], which is also certainly responsible for the decline in measles antibodies from the age of 12 years. Therefore, catch-up measles vaccination for this population seems to be mandatory.

Following the introduction of universal hepatitis B (HBV) vaccination in Ukraine, initially vaccination rates were high but subsequently decreased considerably (92–98% (2004–2007) vs. 21–48% (2010–2016) [30]). We found a serological correlate of protection against hepatitis B in less than 50% in Ukrainian minors ≥ 12 years, considerably lower than in age-matched Germans (84.4%) [33]. Therefore, especially adolescents should be offered HBV vaccination.

### Limitations

In addition to the possible selection bias (like high education and urban background of the Ukrainians included), there are some limitations to our findings. Although our cohort is the largest of its kind, it may not appropriately represent the Ukrainian refugee population. In addition to this, most of our data are survey based, so despite trained interpreters, it is limited in its validity. Furthermore, surveys of parents to assess child health always face the problem that the perception of the child itself might not be reflected. Not all participants were followed up, meaning that it is not possible to report detailed follow-up information. In addition, severity of illness and effects of interrupted medical care on the course of the illness were not recorded since invasion and flight until immigration to Germany.

## Conclusion

Even if most chronic illnesses were as common as in other Western Europe countries, the general health status and well-being of Ukrainian refugee minors was considered to be rather poor. Health care workers in Germany should be aware of this perception.

Collecting information on medical history even with the help of professional interpreters, is limited and seems to insufficiently ensure valid medical data including vaccination status. This was also observed by others [36, 37]. Minor Ukrainian refugees should be screened for tuberculosis, HIV and hepatitis C and B and without documented vaccinations should be vaccinated according to local recommendations (Table 6). More targeted information about healthcare system of the host country (for Germany [38]) and educational campaigns about vaccinations could improve acceptance of healthcare services and reduce vaccination hesitancy.

**Table 6:** Vaccinations recommended for minors. Comparison of vaccination schedules between Ukraine [39] and Germany [28]

Vaccination	Ukraine	Germany
Tetanus	X	X
Diphtheria	X	X
Whooping cough	X	X
Haemophilus influenzae B	X	X
Polio	X	X
Hepatitis B	X	X
Pneumococci		X
Meningococcal C		X
Measles	X	X
Mumps	X	X
Rubella	X	X
Varicella, chickenpox		X
Tuberculosis	X	
Rotavirus		X

## Supplementary Information

Below is the link to the electronic supplementary material.

Supplementary file1 (PDF 1048 KB)

Supplementary file2 (PDF 605 KB)

## Data Availability

No datasets were generated or analysed during the current study.

## Abbreviations

BAMF: Federal Office for Migration and Refugees in Germany
BMI: Body mass index
CDC: Centres for Disease Control and Prevention
CORKID: Study acronym of the study on the seroconversion rate of SARS-CoV-2 in children and adolescents and their parents in the Ruhr area
DGPI: German Society for Paediatric Infectious Diseases
eCRF: Electronic Case Report Form
HIV: Human Immunodeficiency Virus
IgG: Immunoglobulin G
IGRA: Interferon-Gamma-Release Assay
MDR-TB: Multidrug resistant tuberculosis
MMR: Measles Mumps Rubella
Pre-XDR-TB: Pre -extensively drug-resistant tuberculosis
PTSD: Post-traumatic stress disorders
RR-TB: Rifampicin-resistant tuberculosis
SARS-CoV2: Severe acute respiratory syndrome coronavirus type 2
STIKO: Standing Committee on Vaccination at the Robert Koch Institute
TB: Tuberculosis
WHO: World Health Organization
XDR-TB: Extensively drug-resistant tuberculosis
