# Supplementary material for: Health and socio-demographic background of Ukrainian minors and their families in Germany - challenges for refugee medicine: A cross-sectional study from the German Network University Medicine (NUM)
Source: Eur J Pediatr. 2024 Dec 5;184(1):64. doi: 10.1007/s00431-024-05847-2 (PMC11621194; doi:10.1007/s00431-024-05847-2)
Supplement: Supplementary file 1 — Supplementary file1 (PDF 1048 KB) [file 431_2024_5847_MOESM1_ESM.pdf]

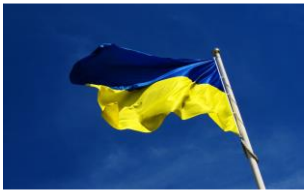

Teil 1 Interview/ Befragung des Hauptprobanden (Erwachsene/“Elternteil“)

Stand: Version 4.1 / Stand 28.07.2022

Anleitung für die Übersetzung/Handhabung des Leitfadens
Gelb markierte Textstellen sind Anleitungen/Hilfen für die Dateneingabe oder werden vom Prüfarzt:in erhoben und müssen **nicht** übersetzt werden!
Grün markierte Textstellen sind Vorerklärungen zu bestimmten Fragen. Diese müssen vor der jeweiligen Frage dem Probanden erklärt werden und daher auch übersetzt werden!
Blau markierte Textstellen sind Instruktionen für den Interviewer:in und müssen übersetzt werden!

Vorbemerkung

In der stress- und konfliktreichen Situation in einer Erstaufnahmeeinrichtung bei gerade geflüchteten und u.U. auch traumatisierten Menschen mit zudem unsicherem Alphabetisierungsgrad bzw. Lesekompetenz ist eine schriftliche Befragung nicht sinnvoll. Die Befragung erfolgt durch zwei oder mehr Personen, von denen eine/ einer ein Arzt/Ärztin ist, ein/e Muttersprachlerin (Dolmetscherin) und eine Hilfskraft zur sofortigen Dateneingabe per Tablet.
Das Vorgehen der Wahl in dieser Situation ist ein semi-strukturiertes Interview mit vorformulierten Fragen, die größtenteils offen formuliert sind. Durch die Übersetzung und gemeinsame fachliche Einordnung der Antworten ist eine valide Zuordnung zu einer oder mehrerer der vorgegebenen Kategorien möglich. Im Falle nicht passender Vorgaben kann an einigen Stellen auch ein Freitext in die Datenbank eingegeben werden. Es sind in vielen Fällen Mehrfachantworten möglich.

Die Datenbank ist in verschiedene Kategorien/Blöcke (siehe Graphik) unterteilt. Die Wechsel zum nächsten Block sind in diesem Leitfaden markiert.

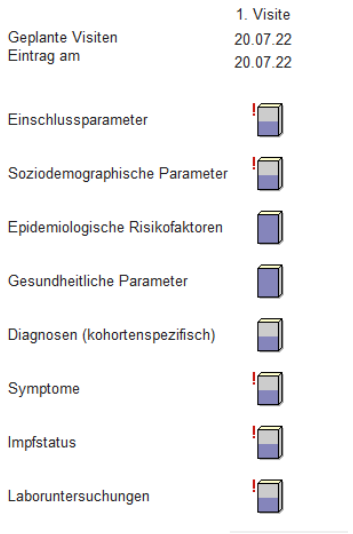

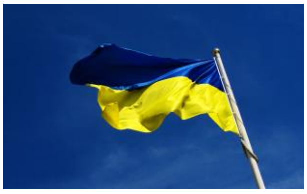

Zunächst werden die persönlichen Daten erhoben und die Einverständniserklärung eingegeben:

Name
Vorname
Geburtsdatum
Geburtsort
Aktuelle Adresse
Telefonnummer (Handy)
Emailadresse

ID: wird vergeben

Als nächstes erfolgt der Wechsel zur Kategorie **„Einschlussparameter“**. Hier werden zunächst einige Eckdaten für jeden Studienteilnehmer eingegeben.

*Die ersten Fragen ergeben meist sich automatisch und müssen **bis auf Frage 5 nicht** erfragt werden.*
*Der Hauptproband ist im Falle von Familien die Sorgeberechtigte Person. Die Frage 3 wird **nicht** an die Interviewten gestellt, sondern vorab vom Prüfarzt:in festgelegt.*

1. **Einschlusszentrum**
2. **Einschlusskohorte**
   AWK Pädiatrie, Erwachsene
3. **Sind Sie der Hauptproband der Gruppe?**
   AWK Ja/Nein
4. **In welcher Sprache wurde die Befragung durchgeführt?**
   AWK deutsch/ukrainisch/russisch/englisch/andere
5. **Wie haben Sie von der Studie erfahren?**
   AWK Flyer/vom behandelnden Arzt/Freunde oder Familie/Social Media/andere

Als nächstes erfolgt der Wechsel zur Kategorie „**Sozialdemographische Parameter“**

1. **Geburtsdatum**
2. **Geschlecht**
   AWK weiblich/männlich/divers/unbestimmt/keine Antwort
3. **Geburtsland**
   AWK Ukraine/anderes Land
   3.1. Wenn das Geburtsland von der Ukraine abweicht Auswahl des Geburtslandes über eine Drop-down-Liste

Nun folgen einige Fragen zum Herkunftsland, die pro Familie/Fluchtgruppe nur einmal von dem Hauptprobanden beantwortet werden müssen!

4. **In welchem Land haben Sie vor der Flucht gelebt?**
   AWK Ukraine/anderes Land
   4.1. Wenn das Land von der Ukraine abweicht Auswahl über eine Drop-down-Liste
   4.2. In welchem Gebiet haben Sie vor der Flucht gelebt?
   Antwort: Gebiete zeigen auf Karte (Folie), Drop-Down-Auswahl

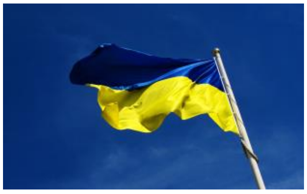

4.3. **Wie groß war der Ort in dem Sie vor Ihrer Flucht gelebt haben?**
AWK: < 1000, 1000-5000, 5000-10.000, 10.000-500.000, >500.000

5. **Seit wann sind Sie in Deutschland**
   Datumsabfrage

6. **Besaßen Sie vor der Flucht ein eigenes Haus oder eine eigene Wohnung (einschließlich Hypothek)?**
   AWK ja/nein
7. **Besaßen Sie vor der Flucht ein funktionierendes Kraftfahrzeug (PKW, LKW, Transporter)?**
   AWK ja/nein
8. **Welchen Schulabschluss haben Sie?**
   AWK: primary, secondary, senior, PTU, Tekhnicum, Universität
9. **Wie sind die Ihre Deutschkenntnisse (Selbsteinschätzung)?**
   AWK: sehr gut, gut, mittelmäßig, schlecht, keine relevanten
10. **Wie war Ihr Erwerbsstatus vor der Flucht?**
    AWK: erwerbstätig, in Ausbildung inkl Schule, in Rente, nicht erwerbstätig

Nun folgen einige Fragen zur Wohn- und Fluchtsituation, die pro Familie/Fluchtgruppe nur einmal von dem Hauptprobanden beantwortet werden müssen!

Erklärung vorweg: wir fragen nach der Wohnung, weil wir das Risiko für Infektionen abschätzen wollen

11. **Wurden die Fragen zur Wohnsituation bereits von einer anderen Person der Gruppe beantwortet?**
    AWK ja/nein
    Wenn 12 = nein
    11.1. **Wie viele Menschen außer Ihnen leben in Ihrem Haushalt (Eltern, Großeltern, Kinder)?**
    Freitext
    11.2. **Wie viele sind davon Kinder/Jugendliche?**
    Freitext
    11.3. **Wie viele Räume hat Ihre Wohnung/das Haus (außer Küche oder Hausflur)?**
    Freitext
12. **Wurden die Fragen zur Fluchtsituation bereits von einer anderen Person der Gruppe beantwortet?**
    AWK ja/nein
    Wenn 13 = nein
    12.1. **Wie lange dauerte die Flucht?**
    Freitext
    12.2. **Und wann startete diese?**
    Datumsabfrage
    12.3. **Womit sind Sie geflohen?**
    AWK Auto, Bahn, Bus, Flugzeug
    12.4. **Mussten Sie während der Flucht mit vielen Menschen außerhalb Ihrer Familie zusammenleben?**
    AWK: ja/nein/unsicher/keine Antwort
    Wenn 13.4.= ja:
    12.4.1. **Wie viele?**
    AWK: < 10, 10-50, 50-100, >100

Als nächstes erfolgt der Wechsel zur Kategorie „**Epidemiologische Risikofaktoren**“

1. **Hatten Sie schon Masern?**
   AWK ja /nein/unsicher
2. **Hatten Sie schon Windpocken?**
   AWK ja /nein/unsicher

Nun folgen einige Fragen zur Tuberkulose und SARS-CoV2, die pro Familie/Fluchtgruppe nur einmal von dem Hauptprobanden beantwortet werden müssen!

Tuberkulose

3.0 Ist in ihrer Familie jemand (Kind, Eltern Geschwistern oder Großeltern) in den letzten 2 Jahren an Tuberkulose erkrankt?

AWK ja/nein/unsicher

3.1.1 Wer war die erste an Tuberkulose erkrankte Person in Ihrer Familie?

AWK Mutter/Vater
Schwester/Bruder
Tochter/Sohn
Großmutter/Großvater
Tante/Onkel
andere

3.1.2. Ist diese Person Teil Ihrer Fluchtgruppe?

AWK ja /nein/ unsicher

3.1.3. Gab es einen Wechsel wegen Unwirksamkeit (Resistenz) der Therapie? Gab es zum Beispiel Spritzen statt nur Tabletten?

AWK ja /nein/ unsicher

3.1.4. Ist die Behandlung zu Ende?

AWK ja/nein/unbekannt

SARS-CoV2

3.2 Ist in ihrer Familie (Kind, Eltern, Geschwistern oder Großeltern) jemand an Corona /SARS-CoV2 erkrankt gewesen?

AWK ja /nein/unsicher
Wenn 3.2. = ja

3.2.1 Ist jemand wegen Corona in einem Krankenhaus stationär gewesen?

AWK ja/nein/unsicher

3.2.2. Ist jemand in ihrer Familie an Corona gestorben?

AWK Ja, Nein, weiß ich nicht

Nun erfolgt der Wechsel zur Kategorie **„Diagnosen (Erwachsene)“**

Haben Sie eine dauerhafte (chronische) Krankheit wie zum Beispiel eine Herz-Kreislauferkrankung, Zuckerkrankheit, oder andere Krankheit?

Die Krankheiten einzeln mittels Folie erfragen

Die Angaben auf der Begleitfolie werden vom Interviewer übertragen.

Wenn auf der Folie keine Krankheiten angekreuzt sind, wird zu „Symptomen“ gewechselt.

Nur wenn auf der Folie Krankheiten angekreuzt sind, wird jeweils nach Medikamenten gefragt.

Haben Sie….?
AWK ja/nein/unsicher

1. Chronische Hepatitis (Leberentzündung)

1.1. **Wenn ja, handelt es sich um eine chronische Hepatitis B?**

1.1.1. **Wann wurde die erstmalig festgestellt (Erstdiagnose)?**
Datumseingabe

1.1.2. Nehmen Sie Medikamente dafür?
AWK ja/nein/unsicher

1.2. **Wenn ja, handelt es sich um eine chronische Hepatitis C?**

1.2.1. **Wann wurde die erstmalig festgestellt (Erstdiagnose)?**
Datumseingabe

1.2.2. Nehmen Sie Medikamente dafür?
AWK ja/nein/unsicher

1.3. **Wenn ja, handelt es sich um eine andere chronische Hepatitis?**

2. HIV/AIDS
AWK ja/nein/weiß ich nicht
Wenn 2= ja

2.1. Wann wurde die Infektion mit HIV zum ersten Mal festgestellt (Erstdiagnose)?
Datumseingabe

2.2. **Bekommen Sie Medikamente gegen *HIV?***
AWK ja/nein/weiß ich nicht

3. Tuberkulose
Wenn 3. = ja

3.1. War es eine Tuberkulose der Lunge oder an anderer oder mehreren Stellen des Körpers (pulmonal oder extrapulmonal)?
AWK: pulmonal/extrapulmonal/disseminiert/weiß ich nicht

3.2. **Wann wurde die Tuberkulose zum ersten Mal festgestellt (Erstdiagnose)?**
Datumseingabe

3.3. **Haben Sie eine spezielle Therapie gegen Tuberkulose bekommen?**
AWK ja/nein/weiß ich nicht

3.4. Wurden andere Medikamente als die Standardtherapie genutzt (hierzu zählen Isoniazid, Rifampicin, Ethambutol und Pyrazinamid)?
AWK ja/nein/weiß ich nicht

3.5. Wurde eine Unwirksamkeit (Resistenz) gegen bestimmte Tuberkulose-Medikamente festgestellt? Gab es zum Beispiel auch Spritzen statt nur Tabletten?
AWK ja/nein/weiß ich nicht

3.6. Wurde die Therapie abgeschlossen/offiziell beendet?
AWK ja/nein/weiß ich nicht

3.7. Wurde die Therapie während/wegen der Flucht unterbrochen?
AWK ja/nein/weiß ich nicht

4. Herz-Kreislauferkrankungen
Wenn 4.= ja

4.1. Bekommen Sie dafür Medikamente?
AWK: ja/nein/unsicher

5. Chronische Lungenerkrankung
Wenn 5.= ja

5.1. Bekommen Sie dafür Medikamente?
AWK: ja/nein/unsicher

6. Chronische Nierenerkrankung
Wenn 6.= ja

6.1. Bekommen Sie dafür Medikamente?
AWK: ja/nein/unsicher

7. **Rheumatologische/immunologische Erkrankungen**
   Wenn 7.= ja
   7.1. Bekommen Sie dafür Medikamente?
   AWK: ja/nein/unsicher
8. **Zuckerkrankheit (Diabetes mellitus)**
   Wenn 8.= ja
   8.1. Bekommen Sie dafür eine Therapie mit Insulin?
   AWK: ja/nein/unsicher
9. **Krebserkrankung (solide Tumorerkrankung)**
   Wenn 9.= ja
   9.1. Bekommen Sie dafür Medikamente?
   AWK: ja/nein/unsicher
10. **hämatoonkologische Erkrankung (Leukämie/Blutkrebs)**
   Wenn 10.= ja
   10.1. Bekommen Sie dafür Medikamente?
   AWK: ja/nein/unsicher
11. **Chronische neurologische Erkrankung**
   Wenn 11.= ja
   11.1. Bekommen Sie dafür Medikamente?
   AWK: ja/nein/unsicher
12. **Psychische Erkrankung**
   Wenn 12.= ja
   12.1. Bekommen Sie dafür Medikamente?
   AWK: ja/nein/unsicher
13. **Frauen im gebärfähigen Alter: Besteht eine Schwangerschaft?**
   Wenn 13.= ja
   13.1. Wann ist der errechnete Geburtstermin?

Nun erfolgt der Wechsel zur Kategorie „**Symptome**“

1. **Wie würden Sie Ihren Gesundheitszustand derzeit beschreiben?**
   AWK ausgezeichnet, sehr gut, gut, weniger gut, schlecht
2. **Hatten Sie in den letzten 3 Monaten eines oder mehrere der hier genannten Krankheitssymptome?**

Die Symptome einzeln mittels Folie erfragen.

Die Angaben auf der Begleitfolie werden vom Interviewer übertragen

AWK: ja, nein

Wenn 2. = ja

Ich habe/leide (einzeln abfragen) unter

2.1 Fieber?
Antwortkategorien ja /nein/unsicher

2.2 Appetitlosigkeit?
Antwortkategorien ja /nein/unsicher

2.3 Schwellung der Lymphknoten?
Antwortkategorien ja /nein/unsicher

2.4 Kopfschmerzen?
Antwortkategorien ja /nein/unsicher

2.5 starkes nächtliches Schwitzen (Nachtschweiß)?
Antwortkategorien ja /nein/unsicher

2.6 ungewünschter Gewichtsverlust?
Antwortkategorien ja /nein/unsicher

2.7 Husten?
Antwortkategorien ja /nein/unsicher
Wenn 2.7. = ja

2.7.1 mit Auswurf?
Antwortkategorien ja /nein/unsicher

2.7.1.1. Farbe des Auswurfs?
AWK: farblos/ gelblich/gelb-grünlich/bräunlich

2.7.1.2. Ist Blut mit dabei (Blutiges Sekret/Hämoptysen)?
Antwortkategorien ja /nein/unsicher

2.8 Kurzatmigkeit (Dyspnoe)?
Antwortkategorien ja /nein/unsicher

2.9. Gastrointestinale Symptome (z.B. Durchfall, Obstipation (Verstopfung), Bauchschmerzen)
Antwortkategorien ja /nein/unsicher

2.10. Haben Sie neurologischen Symptomen (z.B. Lähmung, Gangstörung, Doppelbilder)?
Antwortkategorien ja /nein/unsicher

2.11. Haben Sie noch andere Symptome
Freitext

Nun erfolgt der Wechsel zur Kategorie **„Impfstatus“**

Hinweis: Die Frage nach Impfungen, insbesondere nach Corona-Impfung vorbereiten mit Erklärung:

- *Es gibt generell keine Impfpflicht in Deutschland*
- *Auch für die Corona-Impfung gibt es keine Zwangsimpfung in Deutschland, auch nicht für Jugendliche*
- *Ausnahme: will ein Kind/ Jugendlicher Kindergarten oder Schule besuchen, ist eine Impfung gegen Masern vorgeschrieben*

Wir wollen in dieser Studie nur nach dem Impfstatus fragen, wenn Sie nicht geimpft sind, bieten wir Impfungen an bzw. können diese vermitteln, Impfungen bleiben aber freiwillig

1. Wie ist Ihre allgemeine Einstellung zum Thema „Impfen“?
AWK
1) Ich bin für Impfungen
2)Ich bin für Impfungen, habe aber Sorgen bzw Bedenken gegenüber einzelnen Impfungen
3) Ich bin eher gegen Impfungen
4)Ich habe keine Meinung dazu

Wenn 1. = Antwort 2 (Sorgen bei einzelnen Impfungen)

*1.1.***Ich habe Sorgen/Bedenken gegenüber folgenden Impfungen** *(Mehrfachauswahl möglich)*
AWK SARS-CoV2/Masern/Polio/Andere (Freitext)

Wenn 1= Antwort 1 (für Impfungen)

1.2. **Haben Sie alle Impfungen zur Grundimmunisierung (nach Impfkalender des MOZ) erhalten?**
AWK ja/nein

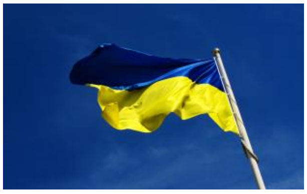

3. **Haben Sie Ihren Impfausweis aus der Ukraine mit**?
   AWK Ja/Nein

4. **Wurden Sie in der Ukraine gegen eine der folgenden Erkrankungen geimpft??**

Die Impfungen einzeln mittels Folie erfragen.

Die Angaben auf der Begleitfolie werden vom Interviewer übertragen

AWK: ja, nein

4.1 Influenza in den letzten 6 Monaten
4.2 Pneumokokken
4.3 Tuberkulose (BCG)
4.4 Wundstarrkrampf (Tetanus)
4.5 Diphterie
4.6 Kinderlähmung (Polio)
4.7 Keuchhusten
4.8 Hepatitis A
4.9 Hepatitis B
4.10 Mumps
4.11 Röteln
4.12 Windpocken
4.13 Meningokokken C
4.14 Haemophilus influenzae Typ b
4.15 Humane Papillomviren (gegen Gebärmutterhalskrebs)

5. **Haben Sie einen Impfpass aus Deutschland für Sich und haben Sie diesen dabei**
   AWK Ja/Nein

6. **Wurden Sie in Deutschland gegen eine oder mehrere dieser Erkrankungen geimpft?**

Die Impfungen einzeln mittels Folie erfragen.

Die Angaben auf der Begleitfolie werden vom Interviewer übertragen

AWK: ja, nein

6.1 Influenza in den letzten 6 Monaten
6.2 Pneumokokken
6.3 Tuberkulose (BCG)
6.4 Wundstarrkrampf (Tetanus)
6.5 Diphterie
6.6 Kinderlähmung (Polio)
6.7 Keuchhusten
6.8 Hepatitis A
6.9 Hepatitis B
6.10 Mumps
6.11 Röteln
6.12 Windpocken
6.13 Meningokokken C
6.14 Haemophilus influenzae Typ b
6.15 Humane Papillomviren (gegen Gebärmutterhalskrebs)

6. **Sind Sie gegen SARS-CoV2 (Corona) geimpft?**
AWK ja/Nein/unsicher

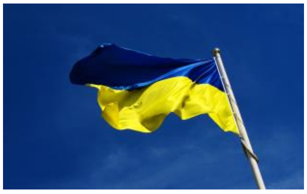

6.1.-4. Mit welchem Wirkstoff wurden Sie bei der 1./2./3./4. Impfung geimpft und in welchem Land?

Die Impfstoffe und das Land der Impfung mittels Folie erfragen.

Die Angaben auf der Begleitfolie werden vom Interviewer übertragen

6.5. Warum sind Sie nicht oder nicht vollständig gegen Corona geimpft?

AWK
Ich kam bisher nicht dazu (z.B. keine Zeit)
Es gab keine Impfstoffe
Ich habe Sorge bzgl. Nebenwirkungen
Ich habe generelle Zweifel an der Sicherheit der Impfstoffe
Ich habe generelle Zweifel an der Wirksamkeit der Impfstoffe
~~*Weil Krankheiten, gegen die man sich impfen lassen kann, kaum noch auftreten*~~
Ich fühle mich nicht ausreichend über die Impfung informiert
Ein Arzt/Ärztin hat mir davon abgeraten
Unsicher/Weiß nicht

Nur wenn die Frage nach der „Masern-Impfung" auf der Folie mit „JA" beantwortet wurde, folgen weitere Fragen!

7. Sind Sie gegen Masern geimpft?

AWK ja/nein/unsicher

7.1. **Wie viele Impfungen haben Sie bereits gegen Masern erhalten?**

Freitext (Zahl)

7.2. **Haben Sie eine oder mehrere Impfungen gegen Masern in Deutschland erhalten?**

AWK Ja/nein/weiß nicht

Nun folgen die ärztliche Untersuchung und der Wechsel zur Kategorie **„Untersuchungsbefund"**

1. Körpergröße in cm

2. Körpergewicht in kg

3. Auskultation Pulmonal

AWK Normalbefund/pathologisch

4. Auskultation Cor

AWK Normalbefund/pathologisch

5. Gefäßstatus

AWK Normalbefund/pathologisch

7. Impfnarbe BCG

AWK ja/nein

8. Hautstatus

AWK Normalbefund/pathologisch

9. Ist eine medizinische Behandlung dringend erforderlich?

AWK ja/nein

10. Verletzungen

Ja/nein/unsicher

10.1. Lokalisation

AWK
Kopf/Hals

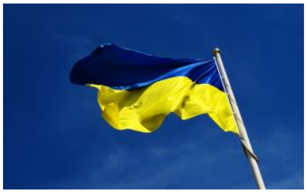

Thorax
Abdomen
Arm/Hand
Bein/Fuß
Mehrere Lokalisationen

10.2. Typ

Oberflächliche Weichteilverletzung
Offene Wunde
Knochenfraktur
Luxation
Nervenverletzung
Gefäßverletzung
Muskel-/Sehnenverletzung
Mehrere Verletzungen

Nun folgt Wechsel zur Kategorie **„Laboruntersuchung“**

1. SARS-CoV2-PCR

AWK: ja, nein, unsicher
Wenn 1 =ja

1.1 Ergebnis qualitativ

AWK negativ/positiv/unklar

1.2 Ergebnis quantitativ

Wert eintragen

1.3 Erfolgte Sequenzierung

AWK ja/nein
Wenn 1.3 = ja

1.3.1 Virusvariante

Drop-Down-Auswahl

2. SARS-CoV2-Antigen-Testung

AWK ja/nein/unsicher
Wenn 2 = ja

2.1 **Testsystem?**

Freitext

2.2 **Ergebnis?**

AWK positiv, kein Erregernachwies, unklar

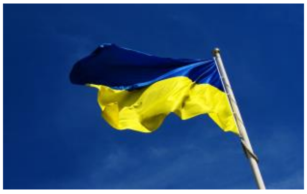

Teil 2
Studienablauf

Die folgenden Fragen beziehen sich auf den Studienablauf und sollten erst nach der vollständigen Dokumentation und Prüfung der Angaben beantwortet werden.

1. Die Studienärzt:innen haben die Befunde in Centraxx gesichtet und beurteilt.
AWK ja/nein
Wenn 1 = ja
1.1 Datumseingabe

2. Wurde der Proband:in eine weitere Behandlung angeboten?
AWK ja/nein

3. Wurde der Proband:in der Befund mitgeteilt?
AWK ja/nein

Wenn 3 = nein
3.1 Warum nicht?
AWK nicht erreicht/unauffälliger Befund

Wenn 3 = ja
3.2 Wie wurde die Information übermittelt?
AWK persönlich/per Telefon/per Fax/per Email/postalisch

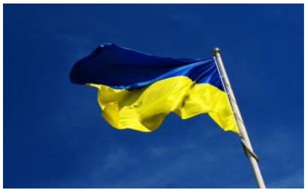

Teil 1 Interview/ Befragung von Erwachsenen (NICHT Hauptproband)
Stand: Version 4.1 / Stand 28.07.2022

Anleitung für die Übersetzung/Handhabung des Leitfadens
Gelb markierte Textstellen sind Anleitungen/Hilfen für die Dateneingabe oder werden vom Prüfarzt:in erhoben und müssen **nicht** übersetzt werden!
Grün markierte Textstellen sind Vorerklärungen zu bestimmten Fragen. Diese müssen vor der jeweiligen Frage dem Probanden erklärt werden und daher auch übersetzt werden!
Blau markierte Textstellen sind Instruktionen für den Interviewer:in und müssen übersetzt werden!

Vorbemerkung
In der stress- und konfliktreichen Situation in einer Erstaufnahmeeinrichtung bei gerade geflüchteten und u.U. auch traumatisierten Menschen mit zudem unsicherem Alphabetisierungsgrad bzw. Lesekompetenz ist eine schriftliche Befragung nicht sinnvoll. Die Befragung erfolgt durch zwei oder mehr Personen, von denen eine/ einer ein Arzt/Ärztin ist, ein/e Muttersprachlerin (Dolmetscherin) und eine Hilfskraft zur sofortigen Dateneingabe per Tablet.
Das Vorgehen der Wahl in dieser Situation ist ein semi-strukturiertes Interview mit vorformulierten Fragen, die größtenteils offen formuliert sind. Durch die Übersetzung und gemeinsame fachliche Einordnung der Antworten ist eine valide Zuordnung zu einer oder mehrerer der vorgegebenen Kategorien möglich. Im Falle nicht passender Vorgaben kann an einigen Stellen auch ein Freitext in die Datenbank eingegeben werden. Es sind in vielen Fällen Mehrfachantworten möglich.

Die Datenbank ist in verschiedene Kategorien/Blöcke (siehe Graphik) unterteilt.
Die Wechsel zum nächsten Block sind in diesem Leitfaden markiert.

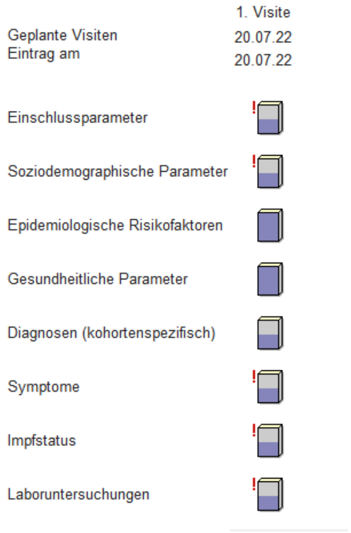

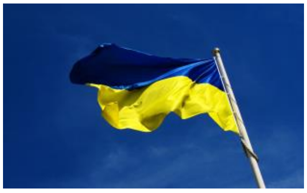

Zunächst werden die persönlichen Daten erhoben und die Einverständniserklärung eingegeben:

Name
Vorname
Geburtsdatum
Geburtsort
Aktuelle Adresse
Telefonnummer (Handy)
Emailadresse

ID: wird vergeben

Als nächstes erfolgt der Wechsel zur Kategorie **„Einschlussparameter“**. Hier werden zunächst einige Eckdaten für jeden Studienteilnehmer eingegeben.

*Die ersten Fragen ergeben meist sich automatisch und müssen **bis auf Frage 5 nicht** erfragt werden.*
*Der Hauptproband ist im Falle von Familien die Sorgeberechtigte Person. Die Frage 3 wird **nicht** an die Interviewten gestellt, sondern vorab vom Prüfarzt:in festgelegt.*

Ist der Proband nicht der Hauptproband muss Frage 3.1. erfragt werden!

1. **Einschlusszentrum**
2. **Einschlusskohorte**
   AWK Pädiatrie, Erwachsene
3. **Sind Sie der Hauptproband der Gruppe?**
   AWK Ja/Nein
   Falls 3. = nein
   3.1. Wie ist die Beziehung zum Hauptprobanden
   AWK
   Mutter/Vater
   Bruder/Schwester
   Großmutter/Großvater
   Tochter/Sohn
   Keine familiäre Beziehung
4. **In welcher Sprache wurde die Befragung durchgeführt?**
   AWK deutsch/ukrainisch/russisch/englisch/andere
5. **Wie haben Sie von der Studie erfahren?**
   AWK Flyer/vom behandelnden Arzt/Freunde oder Familie/Social Media/andere

Als nächstes erfolgt der Wechsel zur Kategorie „**Sozialdemographische Parameter“**

1. **Geburtsdatum**
2. **Geschlecht**
   AWK weiblich/männlich/divers/unbestimmt/keine Antwort
3. **Geburtsland**
   AWK Ukraine/anderes Land

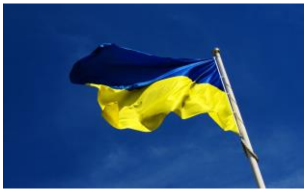

3.1. **Wenn das Geburtsland von der Ukraine abweicht Auswahl des Geburtslandes über eine Drop-down-Liste**

6. **Besaßen Sie vor der Flucht ein eigenes Haus oder eine eigene Wohnung (einschließlich Hypothek)?**
   AWK ja/nein
7. **Besaßen Sie vor der Flucht ein funktionierendes Kraftfahrzeug (PKW, LKW, Transporter)?**
   AWK ja/nein
8. **Welchen Schulabschluss haben Sie?**
   AWK: primary, secondary, senior, PTU, Tekhnicum, Universität
9. **Wie sind die Ihre Deutschkenntnisse (Selbsteinschätzung)?**
   AWK: sehr gut, gut, mittelmäßig, schlecht, keine relevanten
10. **Wie war Ihr Erwerbsstatus vor der Flucht?**
    AWK: erwerbstätig, in Ausbildung inkl Schule, in Rente, nicht erwerbstätig

Als nächstes erfolgt der Wechsel zur Kategorie **„Epidemiologische Risikofaktoren"**

1. **Hatten Sie schon Masern?**
   AWK ja /nein/unsicher
2. **Hatte Sie schon Windpocken?**
   AWK ja /nein/unsicher

Nun erfolgt der Wechsel zur Kategorie **„Diagnosen (Erwachsene)"**

Haben Sie eine dauerhafte (chronische) Krankheit wie zum Beispiel eine Herz-Kreislauferkrankung, Zuckerkrankheit, oder andere Krankheit?

Die Krankheiten einzeln mittels Folie erfragen

Die Angaben auf der Begleitfolie werden vom Interviewer übertragen.

Wenn auf der Folie keine Krankheiten angekreuzt sind, wird zu „Symptomen" gewechselt.

Nur wenn auf der Folie Krankheiten angekreuzt sind, wird jeweils nach Medikamenten gefragt.

Haben Sie….?
AWK ja/nein/unsicher

1. **Chronische Hepatitis (Leberentzündung)**
   1.1. **Wenn ja, handelt es sich um eine chronische Hepatitis B?**
   1.1.1. **Wann wurde die erstmalig festgestellt (Erstdiagnose)?**
   Datumseingabe
   1.1.2. Nehmen Sie Medikamente dafür?
   AWK ja/nein/unsicher
   1.2. **Wenn ja, handelt es sich um eine chronische Hepatitis C?**
   1.2.1. **Wann wurde die erstmalig festgestellt (Erstdiagnose)?**
   Datumseingabe
   1.2.2. Nehmen Sie Medikamente dafür?
   AWK ja/nein/unsicher

1.3. **Wenn ja, handelt es sich um eine andere chronische Hepatitis?**

2. HIV/AIDS

AWK ja/nein/weiß ich nicht

Wenn 2= ja

2.1. Wann wurde die Infektion mit HIV zum ersten Mal festgestellt (Erstdiagnose)?

Datumseingabe

2.2. **Bekommen Sie Medikamente gegen *HIV?***

AWK ja/nein/weiß ich nicht

3. Tuberkulose

Wenn 3. = ja

3.1. War es eine Tuberkulose der Lunge oder an anderer oder mehreren Stellen des Körpers (pulmonal oder extrapulmonal)?

AWK: pulmonal/extrapulmonal/disseminiert/weiß ich nicht

3.2. **Wann wurde die Tuberkulose zum ersten Mal festgestellt (Erstdiagnose)?**

Datumseingabe

3.3. **Haben Sie eine spezielle Therapie gegen Tuberkulose bekommen?**

AWK ja/nein/weiß ich nicht

3.4. Wurden andere Medikamente als die Standardtherapie genutzt (hierzu zählen Isoniazid, Rifampicin, Ethambutol und Pyrazinamid)?

AWK ja/nein/weiß ich nicht

3.5. Wurde eine Unwirksamkeit (Resistenz) gegen bestimmte Tuberkulose-Medikamente festgestellt? Gab es zum Beispiel auch Spritzen statt nur Tabletten?

AWK ja/nein/weiß ich nicht

3.6. Wurde die Therapie abgeschlossen/offiziell beendet?

AWK ja/nein/weiß ich nicht

3.7. Wurde die Therapie während/wegen der Flucht unterbrochen?

AWK ja/nein/weiß ich nicht

4. Herz-Kreislauferkrankungen

Wenn 4.= ja

4.1. Bekommen Sie dafür Medikamente?

AWK: ja/nein/unsicher

5. Chronische Lungenerkrankung

Wenn 5.= ja

5.1. Bekommen Sie dafür Medikamente?

AWK: ja/nein/unsicher

6. Chronische Nierenerkrankung

Wenn 6.= ja

6.1. Bekommen Sie dafür Medikamente?

AWK: ja/nein/unsicher

7. Rheumatologische/immunologische Erkrankungen

Wenn 7.= ja

7.1. Bekommen Sie dafür Medikamente?

AWK: ja/nein/unsicher

8. Zuckerkrankheit (Diabetes mellitus)

Wenn 8.= ja

8.1. Bekommen Sie dafür eine Therapie mit Insulin?

AWK: ja/nein/unsicher

9. Krebserkrankung (solide Tumorerkrankung)

Wenn 9.= ja

9.1. Bekommt Sie dafür Medikamente?

AWK: ja/nein/unsicher

10. hämatoonkologische Erkrankung (Leukämie/Blutkrebs)
Wenn 10.= ja
10.1. Bekommen Sie dafür Medikamente?
AWK: ja/nein/unsicher
11. Chronische neurologische Erkrankung
Wenn 11.= ja
11.1. Bekommen Sie dafür Medikamente?
AWK: ja/nein/unsicher
12. Psychische Erkrankung
Wenn 12.= ja
12.1. Bekommen Sie dafür Medikamente?
AWK: ja/nein/unsicher
13. Frauen im gebärfähigen Alter: Besteht eine Schwangerschaft?
Wenn 13.= ja
13.1. Wann ist der errechnete Geburtstermin?

Nun erfolgt der Wechsel zur Kategorie „**Symptome**“

1. Wie würden Sie Ihren Gesundheitszustand derzeit beschreiben?
AWK ausgezeichnet, sehr gut, gut, weniger gut, schlecht
2. Hatten Sie in den letzten 3 Monaten eines oder mehrere der hier genannten Krankheitssymptome?

Die Symptome einzeln mittels Folie erfragen.
Die Angaben auf der Begleitfolie werden vom Interviewer übertragen
AWK: ja, nein

Wenn 2. = ja
Ich habe/leide (einzeln abfragen) unter
2.1 Fieber?
Antwortkategorien ja /nein/unsicher
2.2 Appetitlosigkeit?
Antwortkategorien ja /nein/unsicher
2.3 Schwellung der Lymphknoten?
Antwortkategorien ja /nein/unsicher
2.4 Kopfschmerzen?
Antwortkategorien ja /nein/unsicher
2.5 starkes nächtliches Schwitzen (Nachtschweiß)?
Antwortkategorien ja /nein/unsicher
2.6 ungewünschter Gewichtsverlust?
Antwortkategorien ja /nein/unsicher
2.7 Husten?
Antwortkategorien ja /nein/unsicher
Wenn 2.7. = ja
2.7.1 mit Auswurf?
Antwortkategorien ja /nein/unsicher
2.7.1.1. Farbe des Auswurfs?
AWK: farblos/ gelblich/gelb-grünlich/bräunlich
2.7.1.2. Ist Blut mit dabei (Blutiges Sekret/Hämoptysen)?

Antwortkategorien ja /nein/unsicher

2.8 Kurzatmigkeit (Dyspnoe)?

Antwortkategorien ja /nein/unsicher

2.9. Gastrointestinale Symptome (z.B. Durchfall, Obstipation (Verstopfung), Bauchschmerzen*)*

Antwortkategorien ja /nein/unsicher

2.10. Haben Sie neurologischen Symptomen (z.B. Lähmung, Gangstörung, Doppelbilder)?

Antwortkategorien ja /nein/unsicher

2.11. Haben Sie noch andere Symptome

Freitext

Nun erfolgt der Wechsel zur Kategorie **„Impfstatus“**

Hinweis: Die Frage nach Impfungen, insbesondere nach Corona-Impfung vorbereiten mit Erklärung:

- *Es gibt generell keine Impfpflicht in Deutschland*
- *Auch für die Corona-Impfung gibt es keine Zwangsimpfung in Deutschland, auch nicht für Jugendliche*
- *Ausnahme: will ein Kind/ Jugendlicher Kindergarten oder Schule besuchen, ist eine Impfung gegen Masern vorgeschrieben*

Wir wollen in dieser Studie nur nach dem Impfstatus fragen, wenn Sie nicht geimpft sind, bieten wir Impfungen an bzw. können diese vermitteln, Impfungen bleiben aber freiwillig

1. Wie ist Ihre allgemeine Einstellung zum Thema „Impfen“?

AWK

1) Ich bin für Impfungen

2)Ich bin für Impfungen, habe aber Sorgen bzw Bedenken gegenüber einzelnen Impfungen

3) Ich bin eher gegen Impfungen

4)Ich habe keine Meinung dazu

Wenn 1. = Antwort 2 (Sorgen bei einzelnen Impfungen)

*1.1.***Ich habe Sorgen/Bedenken gegenüber folgenden Impfungen** *(Mehrfachauswahl möglich)*

AWK SARS-CoV2/Masern/Polio/Andere (Freitext)

Wenn 1= Antwort 1 (für Impfungen)

1.2. **Haben Sie alle Impfungen zur Grundimmunisierung (nach Impfkalender des MOZ) erhalten?**

AWK ja/nein

3. **Haben Sie Ihren Impfausweis aus der Ukraine mit**?

AWK Ja/Nein

4. Wurden Sie in der Ukraine gegen eine der folgenden Erkrankungen geimpft??

Die Impfungen einzeln mittels Folie erfragen.

Die Angaben auf der Begleitfolie werden vom Interviewer übertragen

AWK: ja, nein

4.1 Influenza in den letzten 6 Monaten

4.2 Pneumokokken

4.3 Tuberkulose (BCG)

4.4 Wundstarrkrampf (Tetanus)

4.5 Diphterie

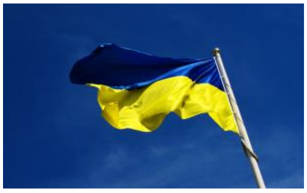

4.6 Kinderlähmung (Polio)
4.7 Keuchhusten
4.8 Hepatitis A
4.9 Hepatitis B
4.10 Mumps
4.11 Röteln
4.12 Windpocken
4.13 Meningokokken C
4.14 Haemophilus influenzae Typ b
4.15 Humane Papillomviren (gegen Gebärmutterhalskrebs)

5. Haben Sie einen Impfpass aus Deutschland für Sich und haben Sie diesen dabei?
AWK Ja/Nein

6. Wurden Sie in Deutschland gegen eine oder mehrere dieser Erkrankungen geimpft?

Die Impfungen einzeln mittels Folie erfragen.

Die Angaben auf der Begleitfolie werden vom Interviewer übertragen

AWK: ja, nein

6.1 Influenza in den letzten 6 Monaten
6.2 Pneumokokken
6.3 Tuberkulose (BCG)
6.4 Wundstarrkrampf (Tetanus)
6.5 Diphterie
6.6 Kinderlähmung (Polio)
6.7 Keuchhusten
6.8 Hepatitis A
6.9 Hepatitis B
6.10 Mumps
6.11 Röteln
6.12 Windpocken
6.13 Meningokokken C
6.14 Haemophilus influenzae Typ b
6.15 Humane Papillomviren (gegen Gebärmutterhalskrebs)

6. Sind Sie gegen SARS-CoV2 (Corona) geimpft?
AWK ja/Nein/unsicher
6.1.-4. Mit welchem Wirkstoff wurden Sie bei der 1./2./3./4. Impfung geimpft und in welchem Land?

Die Impfstoffe und das Land der Impfung mittels Folie erfragen.

Die Angaben auf der Begleitfolie werden vom Interviewer übertragen

6.5. Warum sind Sie nicht oder nicht vollständig gegen Corona geimpft?
AWK
Ich kam bisher nicht dazu (z.B. keine Zeit)
Es gab keine Impfstoffe
Ich habe Sorge bzgl. Nebenwirkungen
Ich habe generelle Zweifel an der Sicherheit der Impfstoffe
Ich habe generelle Zweifel an der Wirksamkeit der Impfstoffe
~~*Weil Krankheiten, gegen die man sich impfen lassen kann, kaum noch auftreten*~~
Ich fühle mich nicht ausreichend über die Impfung informiert
Ein Arzt/Ärztin hat mir davon abgeraten
Unsicher/Weiß nicht

Nur wenn die Frage nach der „Masern-Impfung“ auf der Folie mit „JA“ beantwortet wurde, folgen weitere Fragen!

7. **Sind Sie gegen Masern geimpft?**
   AWK ja/nein/unsicher

   7.1. **Wie viele Impfungen haben Sie bereits gegen Masern erhalten?**
   Freitext (Zahl)
   7.2. **Haben Sie eine oder mehrere Impfungen gegen Masern in Deutschland erhalten?**
   AWK Ja/nein/weiß nicht

Nun folgen die ärztliche Untersuchung und der Wechsel zur Kategorie **„Untersuchungsbefund“**

1. **Körpergröße in cm**
2. **Körpergewicht in kg**
3. **Auskultation Pulmonal**
   AWK Normalbefund/pathologisch
4. **Auskultation Cor**
   AWK Normalbefund/pathologisch
   6. **Gefäßstatus**
      AWK Normalbefund/pathologisch
7. **Impfnarbe BCG**
   AWK ja/nein
   8. **Hautstatus**

AWK Normalbefund/pathologisch

9. **Ist eine medizinische Behandlung dringend erforderlich?**
   AWK ja/nein
10. **Verletzungen**
    Ja/nein/unsicher
    10.1. Lokalisation
    AWK
    Kopf/Hals
    Thorax
    Abdomen
    Arm/Hand
    Bein/Fuß
    Mehrere Lokalisationen
    10.2. Typ
    Oberflächliche Weichteilverletzung
    Offene Wunde
    Knochenfraktur
    Luxation
    Nervenverletzung
    Gefäßverletzung
    Muskel-/Sehnenverletzung
    Mehrere Verletzungen

Nun folgt Wechsel zur Kategorie **„Laboruntersuchung“**

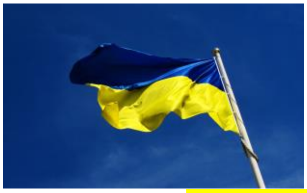

1. SARS-CoV2-PCR

AWK: ja, nein, unsicher

Wenn 1 =ja

1.1 Ergebnis qualitativ

AWK negativ/positiv/unklar

1.2 Ergebnis quantitativ

Wert eintragen

1.3 Erfolgte Sequenzierung

AWK ja/nein

Wenn 1.3 = ja

1.3.1 Virusvariante

Drop-Down-Auswahl

2. SARS-CoV2-Antigen-Testung

AWK ja/nein/unsicher

Wenn 2 = ja

2.1 **Testsystem?**

Freitext

2.2 **Ergebnis?**

AWK positiv, kein Erregernachwies, unklar

Teil 2
Studienablauf

Die folgenden Fragen beziehen sich auf den Studienablauf und sollten erst nach der vollständigen Dokumentation und Prüfung der Angaben beantwortet werden.

1. Die Studienärzt:innen haben die Befunde in Centraxx gesichtet und beurteilt.

AWK ja/nein

Wenn 1 = ja

1.1 Datumseingabe

2. Wurde der Proband:in eine weitere Behandlung angeboten?

AWK ja/nein

3. Wurde der Proband:in der Befund mitgeteilt?

AWK ja/nein

Wenn 3 = nein

3.1 Warum nicht?

AWK nicht erreicht/unauffälliger Befund

Wenn 3 = ja

3.2 Wie wurde die Information übermittelt?

AWK persönlich/per Telefon/per Fax/per Email/postalisch

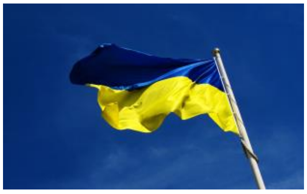

Teil 1 Interview/ Befragung von Familien – „Kinderteil“
Stand: Version 4.2 / Stand 28.07.2022

Anleitung für die Übersetzung/Handhabung des Leitfadens
Gelb markierte Textstellen sind Anleitungen/Hilfen für die Dateneingabe oder werden vom Prüfarzt:in erhoben und müssen **nicht** übersetzt werden!
Grün markierte Textstellen sind Vorerklärungen zu bestimmten Fragen. Diese müssen vor der jeweiligen Frage dem Probanden erklärt werden und daher auch übersetzt werden!
Blau markierte Textstellen sind Instruktionen für den Interviewer:in und müssen übersetzt werden!

Vorbemerkung
In der stress- und konfliktreichen Situation in einer Erstaufnahmeeinrichtung bei gerade geflüchteten und u.U. auch traumatisierten Menschen mit zudem unsicherem Alphabetisierungsgrad bzw. Lesekompetenz ist eine schriftliche Befragung nicht sinnvoll. Die Befragung erfolgt durch zwei oder mehr Personen, von denen eine/ einer ein Arzt/Ärztin ist, ein/e Muttersprachlerin (Dolmetscherin) und eine Hilfskraft zur sofortigen Dateneingabe per Tablet.
Das Vorgehen der Wahl in dieser Situation ist ein semi-strukturiertes Interview mit vorformulierten Fragen, die größtenteils offen formuliert sind. Durch die Übersetzung und gemeinsame fachliche Einordnung der Antworten ist eine valide Zuordnung zu einer oder mehrerer der vorgegebenen Kategorien möglich. Im Falle nicht passender Vorgaben kann an einigen Stellen auch ein Freitext in die Datenbank eingegeben werden. Es sind in vielen Fällen Mehrfachantworten möglich.

Die Datenbank ist in verschiedene Kategorien/Blöcke (siehe Graphik) unterteilt. Die Wechsel zum nächsten Block sind in diesem Leitfaden markiert.

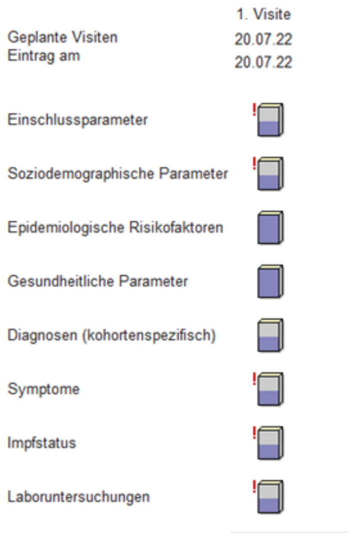

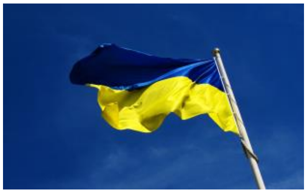

Zunächst werden die Daten dessen, der die Einverständniserklärung unterschreibt, erhoben und die Einverständniserklärung eingegeben:

Name
Vorname
Geburtsdatum
Geburtsort
Aktuelle Adresse
Telefonnummer (Handy)
Emailadresse

ID: wird vergeben

Als nächstes erfolgt der Wechsel zur Kategorie **„Einschlussparameter"**. Hier werden zunächst einige Eckdaten für jeden Studienteilnehmer eingegeben.

Die ersten Fragen ergeben meist sich automatisch und müssen nicht erfragt werden.

1. **Einschlusszentrum**
2. **Einschlusskohorte**
   AWK Pädiatrie, Erwachsene

*Hauptprobanden ist im Falle von Familien die Sorgeberechtigte Person. Die Frage 3 wird **nicht** an die Interviewten gestellt, sondern vorab vom Prüfarzt:in festgelegt.*

*Bei Kindern **muss** daher nur die Frage 3.1. beantwortet werden.*

3. **Sind Sie der Hauptproband der Gruppe?**
   AWK Ja/Nein
   Falls 3. = nein
   3.1. Wie ist die Beziehung zum Hauptprobanden
   AWK
   Mutter/Vater
   Bruder/Schwester
   Großmutter/Großvater
   Tochter/Sohn
   Keine familiäre Beziehung

*Die nächste Frage wird **nicht** an die Interviewten gestellt, sondern ergibt sich von selbst.*

4. **In welcher Sprache wurde die Befragung durchgeführt?**
   AWK deutsch/ukrainisch/russisch/englisch/andere

Die nächste Frage wird bei Kindern übersprungen.

5. **Wie haben Sie von der Studie erfahren?**
   AWK Flyer/vom behandelnden Arzt/Freunde oder Familie/Social Media/andere

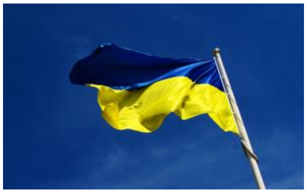

Als nächstes erfolgt der Wechsel zur Kategorie „**Sozialdemographische Parameter**“

1. **Geburtsdatum**
2. **Geschlecht**
   AWK weiblich/männlich/divers/unbestimmt/keine Antwort
3. **Geburtsland**
   AWK Ukraine/anderes Land
   3.1. Wenn das Geburtsland von der Ukraine abweicht Auswahl des Geburtslandes über eine Drop-down-Liste

Als nächstes erfolgt der Wechsel zur Kategorie „**Epidemiologische Risikofaktoren**“

1. **Hatte ihr Kind schon Masern?**
   AWK ja /nein/unsicher
2. **Hatte ihr Kind schon Windpocken?**
   AWK ja /nein/unsicher

Nun erfolgt der Wechsel zur Kategorie **„Diagnosen (Pädiatrie)“**

Hat ihr Kind eine dauerhafte (chronische) Krankheit wie zum Beispiel Asthma, Zuckerkrankheit, oder andere Krankheit?

Die Krankheiten einzeln mittels Folie erfragen

Die Angaben auf der Begleitfolie werden vom Interviewer übertragen.

Wenn auf der Folie keine Krankheiten angekreuzt sind, wird zu „Symptomen“ gewechselt.

Nur wenn auf der Folie Krankheiten angekreuzt sind, wird jeweils nach Medikamenten gefragt.

Hat Ihr Kind…?
AWK ja/nein/unsicher

1. **Angeborene/ererbte Anomalien Krankheiten oder Fehlbildungen (kongenitale Anomalien)**
2. **Entwicklungsverzögerung**
3. **Asthma**
   Wenn 3.= ja
   3.1. Bekommt ihr Kind dafür Medikamente?
   AWK: ja/nein/unsicher
4. **Bronchitis**
   Wenn 4.= ja
   4.1. Bekommt ihr Kind dafür Medikamente?
   AWK: ja/nein/unsicher
5. **Zuckerkrankheit (Diabetes mellitus)**
   Wenn 5.= ja
   5.1. Bekommt ihr Kind eine Therapie mit Insulin?
   AWK: ja/nein/unsicher

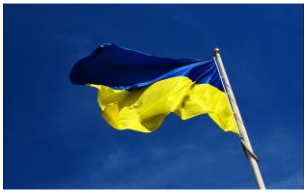

6. Epilepsie (Krampfleiden)
Wenn 6.= ja
6.1. Bekommt ihr Kind dafür Medikamente?
AWK: ja/nein/unsicher

7. Seelisches Leiden
Wenn 7.= ja
7.1. Bekommt ihr Kind dafür Medikamente?
AWK: ja/nein/unsicher

8. Krebserkrankung (solide Tumorerkrankung)
Wenn 8.= ja
8.1. Bekommt ihr Kind dafür Medikamente?
AWK: ja/nein/unsicher

9. hämatoonkologische Erkrankung (Leukämie/Blutkrebs)
Wenn 9.= ja
9.1. Bekommt ihr Kind dafür Medikamente?
AWK: ja/nein/unsicher

10. Bekannte Abwehrschwäche/Immundefekt
Wenn 10.= ja
10.1. Bekommt ihr Kind dafür Medikamente?
AWK: ja/nein/unsicher

11. Tuberkulose
Wenn 11. = ja
11.1. War es eine Tuberkulose der Lunge oder an anderer oder mehreren Stellen des Körpers (pulmonal oder extrapulmonal)?
AWK: pulmonal/extrapulmonal/dissiminiert/weiß ich nicht
***11.2.* Wann wurde die Tuberkulose zum ersten Mal festgestellt (Erstdiagnose)?**
Datumseingabe
***11.3.* Hat ihr Kind eine spezielle Therapie gegen Tuberkulose bekommen?**
AWK ja/nein/weiß ich nicht
11.4. Wurde eine Unwirksamkeit (Resistenz) gegen bestimmte Tuberkulose-Medikamente festgestellt? Gab es zum Beispiel auch Spritzen statt nur Tabletten?
AWK ja/nein/weiß ich nicht
11.5. Wurden andere Medikamente als die Standardtherapie genutzt (hierzu zählen Isoniazid, Rifampicin, Ethambutol und Pyrazinamid)?
AWK ja/nein/weiß ich nicht
11.6. Wurde die Therapie abgeschlossen/offiziell beendet?
AWK ja/nein/weiß ich nicht
11.7. Wurde die Therapie während/wegen der Flucht unterbrochen?
AWK ja/nein/weiß ich nicht

12. HIV/AIDS
AWK ja/nein/weiß ich nicht
Wenn 12= ja
12.1. Wann wurde die Infektion mit HIV zum ersten Mal festgestellt (Erstdiagnose)?
Datumseingabe
12.2.* Bekommt ihr Kind Medikamente gegen *HIV?
AWK ja/nein/weiß ich nicht

Nun erfolgt der Wechsel zur Kategorie „**Symptome**"

1. **Wie würden Sie den Gesundheitszustand Ihres Kindes derzeit beschreiben?**
   AWK ausgezeichnet, sehr gut, gut, weniger gut, schlecht

2. **Litt Ihr Kind in den letzten 3 Monaten unter einem oder mehreren der hier genannten Krankheitssymptome?**
   AWK: ja, nein

Die Symptome einzeln mittels Folie erfragen.

Die Angaben auf der Begleitfolie werden vom Interviewer übertragen

Wenn 2. = ja

Es hat /leidet (einzeln abfragen) unter

2.1 Fieber?
Antwortkategorien ja /nein/unsicher

2.2 Appetitlosigkeit?
Antwortkategorien ja /nein/unsicher

2.3 Schwellung der Lymphknoten?
Antwortkategorien ja /nein/unsicher

2.4 Kopfschmerzen?
Antwortkategorien ja /nein/unsicher

2.5 starkes nächtliches Schwitzen (Nachtschweiß)?
Antwortkategorien ja /nein/unsicher

2.6 ungewünschter Gewichtsverlust?
Antwortkategorien ja /nein/unsicher

2.7 Husten?
Antwortkategorien ja /nein/unsicher
Wenn 2.7. = ja

2.7.1 mit Auswurf?
Antwortkategorien ja /nein/unsicher

2.7.1.1. Farbe des Auswurfs?
AWK: farblos/ gelblich/gelb-grünlich/bräunlich

2.7.1.2. Ist Blut mit dabei (Blutiges Sekret/Hämoptysen)?
Antwortkategorien ja /nein/unsicher

2.8 Kurzatmigkeit (Dyspnoe)?
Antwortkategorien ja /nein/unsicher

2.9. Gastrointestinale Symptome (z.B. Durchfall, Obstipation (Verstopfung), Bauchschmerzen)
Antwortkategorien ja /nein/unsicher

2.10. Leidet ihr Kind unter neurologischen Symptomen (z.B. Lähmung, Gangstörung, Doppelbilder)?
Antwortkategorien ja /nein/unsicher

2.11. Hat ihr Kind noch andere Symptome
Freitext

Die folgenden Fragen bitte mit dieser Erklärung vorbereiten:
„Bei den folgenden Fragen geht es um Ihre eigene Einschätzung, wie es Ihrem Kind insgesamt körperlich und seelisch in dieser schwierigen Situation geht. Die Fragen beziehen auf die Zeit hier in Deutschland nach der Flucht. Es gibt keine richtigen oder falschen Antworten. Wir möchten Ihre ganz persönliche Erfahrung etwas besser verstehen. Dann können wir auch am besten helfen. Wählen Sie spontan die am besten zutreffende Antwort. Wenn Sie eine Frage nicht beantworten möchten, dann können wir diese einfach überspringen"

Instruktion an Interviewer: Wenn sie das Gefühl haben, diese Fragen sind zu belastend, überspringen Sie die Fragen 3 bis 5

3. **Wie würden Sie das seelische Befinden/den seelischen Zustand Ihres Kindes derzeit beschreiben? Dazu gehören auch seine Gefühle, seine Fähigkeit, sich zu konzentrieren und seine Fähigkeit, klar zu denken.**
   Antwortkategorien ausgezeichnet, sehr gut, gut, weniger gut, schlecht
4. **Hat Ihr Kind sich einsam gefühlt**
   Antwortkategorien nie, selten, manchmal, oft, immer
5. **Hat Ihr Kind sich traurig gefühlt?**
   Antwortkategorien nie, selten, manchmal, oft, immer

Nun erfolgt der Wechsel zur Kategorie **„Impfstatus“**

Hinweis: Die Frage nach Impfungen, insbesondere nach Corona-Impfung vorbereiten mit Erklärung:

- *Es gibt generell keine Impfpflicht in Deutschland*
- *Auch für die Corona-Impfung gibt es keine Zwangsimpfung in Deutschland, auch nicht für Jugendliche*
- *Ausnahme: will ein Kind/ Jugendlicher Kindergarten oder Schule besuchen, ist eine Impfung gegen Masern vorgeschrieben*

Wir wollen in dieser Studie nur nach dem Impfstatus fragen, wenn das Kind nicht geimpft ist, bieten wir Impfungen an bzw. können diese vermitteln, Impfungen bleiben aber freiwillig

1. **Wie ist Ihre allgemeine Einstellung zum Thema „Impfen“?**

AWK
1) Ich bin für Impfungen
2)Ich bin für Impfungen, habe aber Sorgen bzw Bedenken gegenüber einzelnen Impfungen
3) Ich bin eher gegen Impfungen
4)Ich habe keine Meinung dazu

Wenn 1. = Antwort 2 (Sorgen bei einzelnen Impfungen)

1.1.
Ich habe Sorgen/Bedenken gegenüber folgenden Impfungen *(Mehrfachauswahl möglich)*
AWK SARS-CoV2/Masern/Polio/Andere (Freitext)

Wenn 1= Antwort 1 (für Impfungen)

1.2. **Hat Ihr Kind alle Impfungen zur Grundimmunisierung (nach Impfkalender des MOZ) erhalten?**
AWK ja/nein

3. **Haben Sie den Impfausweis Ihres Kindes aus der Ukraine mit**?
AWK Ja/Nein

4. **Wurde Ihr Kind in der Ukraine gegen eine der folgenden Erkrankungen geimpft??**

Die Impfungen einzeln mittels Folie erfragen.

Die Angaben auf der Begleitfolie werden vom Interviewer übertragen

AWK: ja, nein

4.1 Influenza in den letzten 6 Monaten
4.2 Pneumokokken
4.3 Tuberkulose (BCG)

4.4 Wundstarrkrampf (Tetanus)
4.5 Diphterie

4.6 Kinderlähmung (Polio)
4.7 Keuchhusten
4.8 Hepatitis A
4.9 Hepatitis B
4.10 Mumps
4.11 Röteln
4.12 Windpocken
4.13 Meningokokken C
4.14 Haemophilus influenzae Typ b
4.15 Humane Papillomviren (gegen Gebärmutterhalskrebs)

5. Haben Sie einen Impfpass aus Deutschland für Ihr Kind und haben Sie diesen dabei
AWK Ja/Nein

6. Wurde ihr Kind in Deutschland gegen eine oder mehrere dieser Erkrankungen geimpft?

Die Impfungen einzeln mittels Folie erfragen.

Die Angaben auf der Begleitfolie werden vom Interviewer übertragen

AWK: ja, nein

6.1 Influenza in den letzten 6 Monaten
6.2 Pneumokokken
6.3 Tuberkulose (BCG)
6.4 Wundstarrkrampf (Tetanus)
6.5 Diphterie
6.6 Kinderlähmung (Polio)
6.7 Keuchhusten
6.8 Hepatitis A
6.9 Hepatitis B
6.10 Mumps
6.11 Röteln
6.12 Windpocken
6.13 Meningokokken C
6.14 Haemophilus influenzae Typ b
6.15 Humane Papillomviren (gegen Gebärmutterhalskrebs)

6. Ist Ihr Kind gegen SARS-CoV2 (Corona) geimpft?
AWK ja/Nein/unsicher

6.1.-4. Mit welchem Wirkstoff wurde Ihr Kind bei der 1./2./3./4. Impfung geimpft und in welchem Land?

Die Impfstoffe und das Land der Impfung mittels Folie erfragen.

Die Angaben auf der Begleitfolie werden vom Interviewer übertragen

6.5. Warum haben Sie Ihr Kind nicht oder nicht vollständig gegen Corona impfen lassen?
AWK
Ich kam bisher nicht dazu (z.B. keine Zeit)
Es gab keine Impfstoffe
Ich habe Sorge bzgl. Nebenwirkungen
Ich habe generelle Zweifel an der Sicherheit der Impfstoffe
Ich habe generelle Zweifel an der Wirksamkeit der Impfstoffe

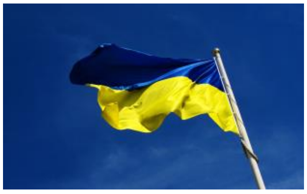

~~*Weil Krankheiten, gegen die man sich impfen lassen kann, kaum noch auftreten*~~
Ich fühle mich nicht ausreichend über die Impfung informiert
Ein Arzt/Ärztin hat mir davon abgeraten
Unsicher/Weiß nicht

Nur wenn die Frage nach der „Masern-Impfung“ auf der Folie mit „JA“ beantwortet wurde, folgen weitere Fragen!

7. **Ist ihr Kind gegen Masern geimpft?**
   AWK ja/nein/unsicher
   7.1. **Wie viele Impfungen hat Ihr Kind bereits gegen Masern erhalten?**
   Freitext (Zahl)
   7.2. **Hat Ihr Kind eine oder mehrere Impfungen gegen Masern in Deutschland erhalten?**
   AWK Ja/nein/weiß nicht

Nun folgen die ärztliche Untersuchung und der Wechsel zur Kategorie **„Untersuchungsbefund“**

1. **Körpergröße in cm**
2. **Körpergewicht in kg**
3. **Auskultation Pulmonal**
   AWK Normalbefund/pathologisch
4. **Auskultation Cor**
   AWK Normalbefund/pathologisch
   6. **HNO-Untersuchung**
   AWK Normalbefund/pathologisch
7. **Impfnarbe BCG**
   AWK ja/nein
8. **Hautstatus**

AWK Normalbefund/pathologisch

9. **Ist eine medizinische Behandlung dringend erforderlich?**
   AWK ja/nein
10. **Verletzungen**
    Ja/nein/unsicher
    10.1. Lokalisation
    AWK
    Kopf/Hals
    Thorax
    Abdomen
    Arm/Hand
    Bein/Fuß
    Mehrere Lokalisationen
    10.2. Typ
    Oberflächliche Weichteilverletzung
    Offene Wunde
    Knochenfraktur
    Luxation
    Nervenverletzung
    Gefäßverletzung
    Muskel-/Sehnenverletzung
    Mehrere Verletzungen

Nun folgt Wechsel zur Kategorie **„Laboruntersuchung“**

1. SARS-CoV2-PCR
AWK: ja, nein, unsicher
Wenn 1 =ja
1.1 Ergebnis qualitativ
AWK negativ/positiv/unklar
1.2 Ergebnis quantitativ
Wert eintragen
1.3 Erfolgte Sequenzierung
AWK ja/nein
Wenn 1.3 = ja
1.3.1 Virusvariante
Drop-Down-Auswahl
2. SARS-CoV2-Antigen-Testung
AWK ja/nein/unsicher
Wenn 2 = ja
2.1 **Testsystem?**
Freitext
2.2 **Ergebnis?**
AWK positiv, kein Erregernachwies, unklar

Teil 2
Studienablauf

Die folgenden Fragen beziehen sich auf den Studienablauf und sollten erst nach der vollständigen Dokumentation und Prüfung der Angaben beantwortet werden.

1. Die Studienärzt:innen haben die Befunde in Centraxx gesichtet und beurteilt.
AWK ja/nein
Wenn 1 = ja
1.1 Datumseingabe
2. Wurde der Proband:in eine weitere Behandlung angeboten?
AWK ja/nein
3. Wurde der Proband:in der Befund mitgeteilt?
AWK ja/nein

Wenn 3 = nein
3.1 Warum nicht?
AWK nicht erreicht/unauffälliger Befund

Wenn 3 = ja
3.2 Wie wurde die Information übermittelt?
AWK persönlich/per Telefon/per Fax/per Email/postalisch
