# Supplementary material for: Health and socio-demographic background of Ukrainian minors and their families in Germany - challenges for refugee medicine: A cross-sectional study from the German Network University Medicine (NUM)
Source: Eur J Pediatr. 2024 Dec 5;184(1):64. doi: 10.1007/s00431-024-05847-2 (PMC11621194; doi:10.1007/s00431-024-05847-2)
Supplement: Supplementary file 2 — Supplementary file2 (PDF 605 KB) [file 431_2024_5847_MOESM2_ESM.pdf]

Text passages that are highlighted in yellow are instructions/assistance for data entry or are collected by the study doctor and don't have to be translated.

Text passages that are highlighted in green are explanations of some questions. These must be explained to the participant and thus need to be translated.

Text passages that are highlighted in blue are instructions for the interviewer and must be translated.

Part 1
Interview - "head" participant (Adults/Parents)
Version 4.1, 28.07.2022

Preliminary remark

In the stressful and conflict-ridden situation in an initial reception centre with people who have just fled and who may also be traumatized and whose literacy or reading skills are also uncertain, a written survey is not practical. The interview is conducted by two or more people, one of whom is a doctor, a native speaker (interpreter) and an assistant for immediate data entry via tablet.
The procedure of choice in this situation is a semi-structured interview with pre-formulated questions, most of which are open-ended. By translating and jointly classifying the answers according to subject, a valid assignment to one or more of the predefined categories is possible. In some cases, free text can also be entered into the database if there are unsuitable specifications. In many cases, multiple answers are possible.
The database is divided into different categories/blocks (see diagram, German version). The changes to the next block are marked in this guide.

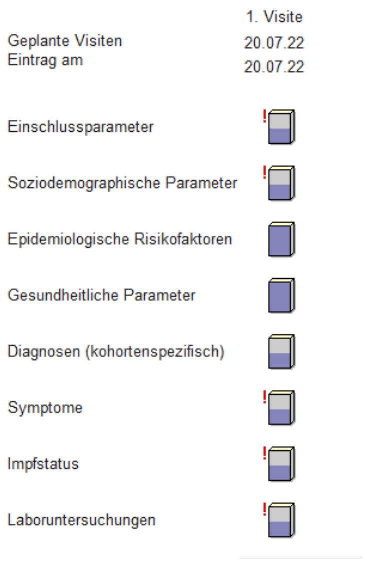

First, the data of the person signing the declaration of consent is collected and the declaration of consent is entered

Surname
Name
Date of birth
Place of birth
Address
Phone number
Email-address

ID: is assigned

The next step is to proceed to the "Inclusion parameters" category. Here you first enter some key data for each study participant

The first questions usually unfold automatically und don't need to, apart from question 5, to be asked.
The "head" participant in case of a family is the custodian. Question 3 is not to be asked the participant, but is answered ex ante by investigator.

1. Center
2. Cohort *(Peadiatric/Adult)*
3. Are you the "head" participant? *(Yes/No)*
4. In which language the interview was conducted?
   (German/Ukrainian/Russian/English/other)
5. Who did you come to know the study?
   (Flyer/from the attending physician/friends or family/social media/other)

The next step is to switch to the "Social demographic parameters" category

1. Date of birth
2. Sex
   Answers: female/male/diverse/undefined
3. Country of birth
   Answers: Ukraine/another country
   3.1 If the country of birth differs from Ukraine, the country is to be selected in a drop-down menu

After that, there are some questions about the native country that must be answered only once per family by the "head" participant.

4. In which country did you live before the flight?
   Answers: Ukraine/another country

4.1 If the country differs from Ukraine, the country is to be selected in a drop-down menu

4.2 In which region did you live before the flight?
Answers: Show regions on card (sheet), dorp-down-selection

4.3 How much inhabitants have the place you have lived before?
Answers: <1000, 1000-5000, 5000-10000, 10000-500000, >500000

5. How long are you in Germany?
   Ask for date
6. Did you own a house or a own flat (mortgage included) before the flight?
   Answers: yes/no
7. Did you own a functional vehicle (car, van)?
   Answers: yes/no
8. What is your last educational institution graduated?
   Answers: primary, secondary, senior, PTU, Technicum, University
9. How is your knowledge of the german language (self-assessment)?
   Answers: very good, good, average, not good, not relevant
10. How was your activity status before the flight?
    Answers: employed, in education (school included), retired, unemployed

Now some questions regarding the circumstances of living and flighting that must be answered only once per family by the “head” participant

Explanation: we ask for the flat, because we want to approximate the risk for infections.

11. Were the questions regarding the living situation already answered by another person of the group?
    Anwers: yes/no
    If 11 = no

11.1 How many person do live with you (Parents, Grandparents, Children)?
11.2 How many of them are children?
11.3 How many rooms does your flat/house have (kitchen and floor excluded)?

12. Were the questions regarding the flight situation already answered by another person of the group?
    Anwers: yes/no
    If 12 = no

12.1 How long did your flight last?
12.2 When did it start?
Ask for date
12.3 How did you flee?
Anwers: by car, train, bus, plane

12.4 Did you need to live with other people apart from your family during the flight?
Answers: yes/no/uncertain/no answer
If 12.4 = yes
12.4.1 How many?
Answers: <10, 10-50, 50-100,>100

The next step is to switch to the "Epidemiological risk factors" category

1. Have you had measles?
   Answers: yes/no/uncertain
2. Have you had varicella?
   Answers: yes/no/uncertain

Now, some questions concerning tuberculosis and SARS-CoV2 to be answered by the “head” participant only

Tuberculosis
3.0 Has anybody in your family (Child, Parents, Grandparents) suffered from tuberculosis in the past 2 years?
Answers: yes/no/uncertain
3.1.1. Who was the first person in your family suffering from tuberculosis?
Answers: Mother/father
Sister/brother
Daughter/son
Grandmother/grandfather
Aunt/uncle
others
3.1.2. Is that person part of your flight group?
Answers: yes/no/uncertain
3.1.3. Was there a change of therapy due to a restistency? Were there injections than just pills?
Answers: yes/no/uncertain
3.1.4. Is the therapy already terminated?
Answers: yes/no/uncertain

SARS-CoV2
3.2 Has anybody in your family had Covid?
Answers: yes/no/uncertain
If 3.2. = yes
3.2.1. Was someone in hospital because of it?
Answers: yes/no/uncertain
3.2.2. Did anybody in your family because of Corona?
Answers: yes/no/uncertain

Now go to the "Diagnoses (adults)" category

Do you have a persistent (chronic) disease like diabetes, cardiovascular diseases or others?

Ask for the all the diseases using the sheet

The information on the accompanying slide is transferred by the interviewer

If no diseases are ticked on the slide, switch to "Symptoms".<br>Only if diseases are marked, it must be asked for drugs

Do you have....?
Answers: yes/no/uncertain

1 Chronic hepatitis
- 1.1 If yes, is it a chronic hepatitis B?
  - 1.1.1 When was the diseases diagnosed firstly?
    Enter a date
  - 1.1.2 Do you take any drugs?
    Answers: yes/no/uncertain
- 1.2. If yes, is it a chronic hepatitis C?
  - 1.2.1 When was the diseases diagnosed firstly?
    Enter a date
  - 1.2.2 Do you take any drugs?
    Answers: yes/no/uncertain
- 1.3. If yes, is it another form of chronic hepatitis?

2 HIV/AIDS
Answers: yes/no/I don’t know
If 2 = yes

- 2.2 When was the diseases diagnosed firstly?
  Enter a date
- 2.2 Do you take drugs against HIV?
  Answers: yes/no/uncertain

3 Tuberculosis
If 3. = yes
- 3.1. Was it a tuberculosis of the lung or at another location (pulmonary or extrapulmonary)?
  Answers: pulmonary/extrapulmonary/disseminated/uncertain
- 3.2 When was it diagnosed for the first time?
  Enter a date
- 3.3 Did you get a special therapy against tuberculosis?
  Answers: yes/no/uncertain

3.4 Where other drugs than the standard therapy used (Isozianid, Rifampicin, Ethambutanol, Pyrazinamid)?
Answers: yes/no/uncertain
3.5 Was a restistency against some tuberculosis-drugs constated?
Answers: yes/no/uncertain
3.6 Was the therapy officially terminated?
Answers: yes/no/uncertain
3.7 Was the therapy interrupted during the flight?
Answers: yes/no/I don't know

4 Cardiovascular diseases
If 4. = yes
4.1 Do you get drugs for it?
Answers: yes/no/uncertain
5 Chronic pulmonary diseases
If 5. = yes
5.1 Do you get drugs for it?
Answers: yes/no/uncertain
6 Chronic diseases of the kidney?
If 6. = yes
6.1 Do you get drugs for it?
Answers: yes/no/uncertain
7 Rheumatologic/immunologic diseases
If 7. = yes
7.1 Do you get drugs for it?
Answers: yes/no/uncertain
8 Diabetes mellitus
If 8. = yes
8.1 Do you get a therapy with insulin?
Answers: yes/no/uncertain
9 Cancer (solid tumor)
If 9. = yes
9.1 Do you get drugs for it?
Answers: yes/no/uncertain
10 Hematooncologic diseases
If 10. = yes
10.1 Do you get drugs for it?
Answers: yes/no/uncertain
11 Chronic neurologic diseases
If 11. = yes
11.1 Do you get drugs for it?
Answers: yes/no/uncertain
12 Psychic diseases
If 12. = yes
12.1 Do you get drugs for it?
Answers: yes/no/uncertain
13 Women in reproductive age: Are you pregnant?
If 13. = yes

13.1 What is the calculated date of birth?

Now switch to the "Symptoms" category

1. How would you describe the general status of your health?
   Answers: excellent/very good/good/less good/bad

2. Have you had any of the mentioned symptoms in the past 3 months?

Ask for the symptoms using the sheet

The information on the accompanying slide is transferred by the interviewer

Answers: yes, no

If 2. = yes

I have/I suffer from (ask separately)

2.1 Fever?
Answers: yes/no/uncertain

2.2 Loss of appetite?
Answers: yes/no/uncertain

2.3 Sweeling of lymph nodes?
Answers: yes/no/uncertain

2.4 Headache?
Answers: yes/no/uncertain

2.5 Night sweat?
Answers: yes/no/uncertain

2.6 Loss of weight?
Answers: yes/no/uncertain

2.7 Cough?
Answers: yes/no/uncertain

2.7.1 With secretion?
Answers: yes/no/uncertain

2.7.1.1 Color of the secretion?
Answer: Clear/yellowish/yellow-greenish/brownish

2.7.1.2 Is the secretion consisting of blood?
Answers: yes/no/uncertain

2.8. Short breath (dyspnea)?
Answers: yes/no/uncertain

2.9. Gastrointestinal symptoms (e.g. diarrhea, constipation, stomachache)?
Answers: yes/no/uncertain

2.10. Neurologic symptoms (e.g. paralysis, ataxia, diplopic images)?
Answers: yes/no/uncertain

2.11. Other symptoms?

Now switch to the "Vaccination status" category

Note: Prepare the questions for vaccination, especially corona-vaccination with explanation

- There is no general duty for vaccination in Germany
- Even for corona there no duty, also not for adolescents
- Exception: if a child/adolescent wanst to go to kindergarten or school, vaccination against measles is required

In this study, we would like to ask for your status of vaccination. If you are not immunized, we do offer or conciliate vaccination. Vaccination remains voluntarily.

1. What is your general attitude towards vaccination?
   1) I support vaccination
   2) I support vaccination, but have worries concerning some vaccination
   3) I am against vaccination
   4) I don't have an opinion on this

If 1 = Answer 2 (worries regarding special vaccination)

1.1 I have worries concerning the following vaccination
    Answers: SARS-CoV2/measles/polio/others

If 1 = Answer 1 (for vaccination)

1.2 Did you get all the vaccination for basic immunization (according to the recommendation of MOZ)?
    Answers: yes/no

2. Did you bring your vaccination card from Ukraine?
   Answers: yes/no
3. Were you vaccinated against one of the following diseases in Ukraine?

Ask for each vaccination using the sheet
The information on the accompanying slide is transferred by the interviewer

Answers: yes, no

Influenza in the past 6 months

Pneumococcus

Tuberculosis

Tetanus

Diphtheria

Poliomyelitis

Whooping cough (Pertussis)

Hepatitis A

Hepatitis B

Mumps

Rubella

Varicella

Meningococcus C

Haemophilus influenzae type b

Human papilloma virus (cervical carcinoma)

4. Do you have a German vaccination card and do you have it with you?
   Answers: yes/no

5. Were you vaccinated against one or several of these diseases in Germany?

Ask for each vaccination using the sheet

The information on the accompanying slide is transferred by the interviewer

Answers: yes, no

Influenza in the past 6 months

Pneumococcus

Tuberculosis

Tetanus

Diphtheria

Poliomyelitis

Whooping cough (Pertussis)

Hepatitis A

Hepatitis B

Mumps

Rubella

Varicella

Meningococcus C

Haemophilus influenzae type b

Human papilloma virus (cervical carcinoma)

6. Are you vaccinated against SARS-CoV2?
   Answer: yes/no
   6.1-4. What vaccine did you get at the 1./2./3./4. Vaccination and in which country?

Ask for vaccinations and the country of vaccination using the sheet

The information on the accompanying slide is transferred by the interviewer

6.5. Why are you not/not fully vaccinated against corona?

Answers:

I didn't have time for it

There were no vaccines available

I have worries concerning secondary effects

I have general doubts regarding the security of vaccines

I have general doubts regarding the efficacy of vaccines

I don't feel adequately informed on the vaccination

A doctor advised me against vaccination

Uncertain/I don't know

Only if the question for measles vaccination on sheet 14 is answered positively, other questions follow.

7. Are you vaccinated against measles?
   Answers: yes/no/uncertain

7.1 How many vaccinations have you already gotten?
7.2 Did you get one or several vaccinations against measles in Germany?
Answers: yes/no/uncertain

This is followed by the medical examination and the change to the category "Examination findings"

1. height in cm
2. body weight in kg
3. auscultation pulmonary
normal findings/pathological
4. auscultation cor
Normal findings/pathological
5. vascular status
Normal findings/pathological
7. vaccination scar BCG
yes/no
8. skin status
Normal findings/pathological
9. is medical treatment urgently required?
yes/no
10. injuries
Yes/no/uncertain
10.1 Localization
Head/neck
Thorax
Abdomen
Arm/hand
Leg/Foot
Multiple localizations
10.2 Type
Superficial soft tissue injury
Open wound
Bone fracture
Dislocation
Nerve injury
Vascular injury
Muscle/tendon injury
Multiple injuries

Now switch to the category **"Laboratory examination"**

1. SARS-CoV2-PCR
yes, no, uncertain
If 1 = yes

1.1 Qualitative result
negative/positive/unclear
1.2 Quantitative result
Enter value
1.3 Successful sequencing
yes/no
If 1.3 = yes
1.3.1 Virus variant
Drop-down selection
Free text
2. SARS-CoV2 antigen test
yes/no/uncertain
If2=yes
2.1 **Test system?**
2.2 **Result?**
positive, no pathogen detection, unclear

Part 2
Study procedure

The following questions relate to the study procedure and should only be answered after the complete documentation and examination of the data.

1 The study physicians have reviewed and assessed the findings in Centraxx.
yes/no
If1=yes
1.1 Date entry
2. was the participant offered further treatment?
yes/no
3. was the participant informed of the findings?
yes/no
If 3 = no
3.1 Why not?
If 3 = yes
not reached/conspicuous findings
3.2 How was the information communicated?
in person/by telephone/fax/by email/by post

Part 1
Interview of adults (not "head" participant)
Version 4.1, 28.07.2022

Preliminary remark

In the stressful and conflict-ridden situation in an initial reception centre with people who have just fled and who may also be traumatized and whose literacy or reading skills are also uncertain, a written survey is not practical. The interview is conducted by two or more people, one of whom is a doctor, a native speaker (interpreter) and an assistant for immediate data entry via tablet.
The procedure of choice in this situation is a semi-structured interview with pre-formulated questions, most of which are open-ended. By translating and jointly classifying the answers according to subject, a valid assignment to one or more of the predefined categories is possible. In some cases, free text can also be entered into the database if there are unsuitable specifications. In many cases, multiple answers are possible.
The database is divided into different categories/blocks (see diagram, German version). The changes to the next block are marked in this guide.

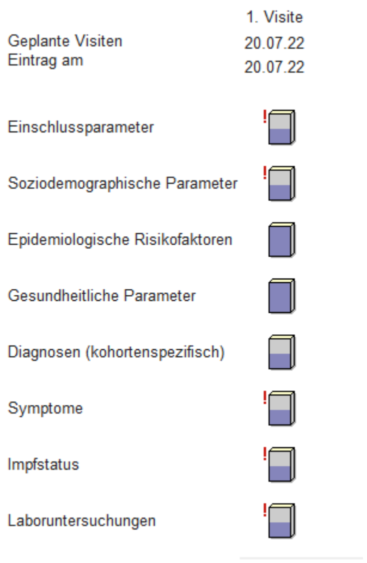

First, the data of the person signing the declaration of consent is collected and the declaration of consent is entered

Surname
Name
Date of birth
Place of birth
Address
Phone number
Email-address

ID: is assigned

The next step is to proceed to the "Inclusion parameters" category. Here you first enter some key data for each study participant

The first questions usually unfold automatically und don't need to, apart from question 5, to be asked.

The "head" participant in case of a family is the custodian. Question 3 is not to be asked the participant, but is answered ex ante by investigator.

If the participant is not the "head" participant, question 3.1 must be asked!

1. Center
2. Cohort *(Peadiatric/Adult)*
3. Are you the "head" participant? *(Yes/No)*
   Answers: yes/no
   If 3. = no
   1.1 What is your relationship to the "head" participant?
      Answers: Mother/father
      Brother/sister
      Grandmother/grandfather
      Daughter/son
      No familiary relationship
4. In which language the interview was conducted?
   (German/Ukrainian/Russian/English/other)
5. Who did you come to know the study?
   (Flyer/from the attending physician/friends or family/social media/other)

The next step is to switch to the "Social demographic parameters" category

1. Date of birth
2. Sex
   Answers: female/male/diverse/undefined
3. Country of birth
   Answers: Ukraine/another country
   3.1. If the country of birth differs from Ukraine, the country is to be selected in a drop-down menu

6. Did you own a house or an own flat (mortgage included) before the flight?
   Answers: yes/no
7. Did you own a functional vehicle (car, van, transporter)?
   Answers: yes/no
8. What is your last educational institution graduated?
   Answers: primary, secondary, senior, PTU, Technicum, university
9. How is your knowledge of the german language (self-assessment)?

Answers: very good, good, average, bad, not relevant

10. How was your activity status before the flight?
    Answers: employed, in education (school included), retired, unemployed

The next step is to switch to the "Epidemiological risk factors" category

1. Have you had measles?
   Answers: yes/no/uncertain
2. Have you had varicella?
   Answers: yes/no/uncertain

Now go to the "Diagnoses (adults)" category

Do you have a persistent (chronic) disease like diabetes, cardiovascular diseases or others?

Ask for the all the diseases using the sheet

The information on the accompanying slide is transferred by the interviewer

If no diseases are ticked on the slide, switch to "Symptoms".
Only if diseases are marked, it must be asked for drugs

Do you have....?
Answers: yes/no/uncertain

1 Chronic hepatitis
  1.2 If yes, is it a chronic hepatitis B?
    1.2.1 When was the diseases diagnosed firstly?
      Enter a date
    1.2.2 Do you take any drugs?
      Answers: yes/no/uncertain
  1.2. If yes, is it a chronic hepatitis C?
    1.2.1 When was the diseases diagnosed firstly?
      Enter a date
    1.2.2 Do you take any drugs?
      Answers: yes/no/uncertain
  1.3. If yes, is it another form of chronic hepatitis?

2 HIV/AIDS
  Answers: yes/no/I don't know
  If 2 = yes

  2.2 When was the diseases diagnosed firstly?
    Enter a date

2.2 Do you take drugs against HIV?
Answers: yes/no/uncertain

3 Tuberculosis
If 3. = yes
3.2. Was it a tuberculosis of the lung or at another location (pulmonary or extrapulmonary)?
Answers: pulmonary/extrapulmonary/disseminated/uncertain
3.2 When was it diagnosed for the first time?
Enter a date
3.3 Did you get a special therapy against tuberculosis?
Answers: yes/no/uncertain
3.4 Where other drugs than the standard therapy used (Isozianid, Rifampicin, Ethambutanol, Pyrazinamid)?
Answers: yes/no/uncertain
3.5 Was a restistency against some tuberculosis-drugs constated?
Answers: yes/no/uncertain
3.6 Was the therapy officially terminated?
Answers: yes/no/uncertain
3.7 Was the therapy interrupted during the flight?
Answers: yes/no/I don't know

4 Cardiovascular diseases
If 4. = yes
4.1 Do you get drugs for it?
Answers: yes/no/uncertain
5 Chronic pulmonary diseases
If 5. = yes
5.1 Do you get drugs for it?
Answers: yes/no/uncertain
6 Chronic diseases of the kidney?
If 6. = yes
6.1 Do you get drugs for it?
Answers: yes/no/uncertain
7 Rheumatologic/immunologic diseases
If 7. = yes
7.1 Do you get drugs for it?
Answers: yes/no/uncertain
8 Diabetes mellitus
If 8. = yes
8.1 Do you get a therapy with insulin?
Answers: yes/no/uncertain
9 Cancer (solid tumor)
If 9. = yes
9.1 Do you get drugs for it?
Answers: yes/no/uncertain

10 Hematooncologic diseases

If 10. = yes

10.1 Do you get drugs for it?

Answers: yes/no/uncertain

11 Chronic neurologic diseases

If 11. = yes

11.1 Do you get drugs for it?

Answers: yes/no/uncertain

12 Psychic diseases

If 12. = yes

12.1 Do you get drugs for it?

Answers: yes/no/uncertain

13 Women in reproductive age: Are you pregnant?

If 13. = yes

13.1 What is the calculated date of birth?

Now switch to the "Symptoms" category

1 How would you describe the general status of your health?

Answers: excellent/very good/good/less good/bad

2 Have you had any of the mentioned symptoms in the past 3 months?

Ask for the symptoms using the sheet

The information on the accompanying slide is transferred by the interviewer

Answers: yes, no

If 2. = yes

I have/I suffer from (ask separately)

2.1 Fever?

Answers: yes/no/uncertain

2.2 Loss of appetite?

Answers: yes/no/uncertain

2.3 Sweeling of lymph nodes?

Answers: yes/no/uncertain

2.4 Headache?

Answers: yes/no/uncertain

2.5 Night sweat?

Answers: yes/no/uncertain

2.6 Loss of weight?

Answers: yes/no/uncertain

2.7 Cough?

Answers: yes/no/uncertain

2.7.1 With secretion?

Answers: yes/no/uncertain

2.7.1.1 Color of the secretion?

Answer: Clear/yellowish/yellow-greenish/brownish

2.7.1.2 Is the secretion consisting of blood?

Answers: yes/no/uncertain

2.8 Short breath (dyspnea)?

Answers: yes/no/uncertain

2.9 Gastrointestinal symptoms (e.g. diarrhea, constipation, stomachache)?

Answers: yes/no/uncertain

2.10 Neurologic symptoms (e.g. paralysis, ataxia, diplopic images)?

Answers: yes/no/uncertain

2.11 Other symptoms?

Now switch to the "Vaccination status" category

Note: Prepare the questions for vaccination, especially corona-vaccination with explanation

- There is no general duty for vaccination in Germany
- Even for corona there no duty, also not for adolescents
- Exception: if a child/adolescent wanst to go to kindergarten or school, vaccination against measles is required

In this study, we would like to ask for your status of vaccination. If you are not immunized, we do offer or conciliate vaccination. Vaccination remains voluntarily.

1. What is your general attitude towards vaccination?
   1) I support vaccination
   2) I support vaccination, but have worries concerning some vaccination
   3) I am against vaccination
   4) I don't have an opinion on this

If 1 = Answer 2 (worries regarding special vaccination)

1.1 I have worries concerning the following vaccination
Answers: SARS-CoV2/measles/polio/others

If 1 = Answer 1 (for vaccination)

1.2 Did you get all the vaccination for basic immunization (according to the recommendation of MOZ)?
Answers: yes/no

2. Did you bring your vaccination card from Ukraine?
   Answers: yes/no
3. Were you vaccinated against one of the following diseases in Ukraine?

Ask for each vaccination using the sheet
The information on the accompanying slide is transferred by the interviewer

Answers: yes, no

Influenza in the past 6 months

Pneumococcus

Tuberculosis

Tetanus

Diphtheria

Poliomyelitis

Whooping cough (Pertussis)

Hepatitis A

Hepatitis B

Mumps

Rubella

Varicella

Meningococcus C

Haemophilus influenzae type b

Human papilloma virus (cervical carcinoma)

4. Do you have a german vaccination card and do you have it with you?
   Answers: yes/no

5. Were you vaccinated against one or several of these diseases in Germany?

   Ask for each vaccination using the sheet
   The information on the accompanying slide is transferred by the interviewer

   Answers: yes, no

   Influenza in the past 6 months

Pneumococcus

Tuberculosis

Tetanus

Diphtheria

Poliomyelitis

Whooping cough (Pertussis)

Hepatitis A

Hepatitis B

Mumps

Rubella

Varicella

Meningococcus C

Haemophilus influenzae type b

Human papilloma virus (cervical carcinoma)

6. Are you vaccinated against SARS-CoV2?
   Answer: yes/no
   6.1-4. What vaccine did you get at the 1./2./3./4. Vaccination and in which country?

Ask for vaccinations and the country of vaccination using the sheet

The information on the accompanying slide is transferred by the interviewer

6.5. Why are you not/not fully vaccinated against corona?

Answers:

I didn't have time for it

There were no vaccines available

I have worries concerning secondary effects

I have general doubts regarding the security of vaccines

I have general doubts regarding the efficacy of vaccines

I don't feel adequately informed on the vaccination

A doctor advised me against vaccination

Uncertain/I don't know

Only if the question for measles vaccination on sheet 14 is answered positively, other questions follow.

7. Are you vaccinated against measles?
   Answers: yes/no/uncertain

7.1 How many vaccinations have you already gotten?

7.2 Did you get one or several vaccinations against measles in Germany?
   Answers: yes/no/uncertain

This is followed by the medical examination and the change to the category "Examination findings"

1. height in cm
2. body weight in kg
3. auscultation pulmonary
normal findings/pathological
4. auscultation cor
Normal findings/pathological
5. vascular status
Normal findings/pathological
7. vaccination scar BCG
yes/no
8. skin status
Normal findings/pathological
9. is medical treatment urgently required?
yes/no
10. injuries
Yes/no/uncertain
10.1 Localization
Head/neck
Thorax
Abdomen
Arm/hand
Leg/Foot
Multiple localizations

10.2 Type
Superficial soft tissue injury
Open wound
Bone fracture
Dislocation
Nerve injury
Vascular injury
Muscle/tendon injury
Multiple injuries

Now switch to the category **"Laboratory examination"**

1. SARS-CoV2-PCR
yes, no, uncertain
If 1 = yes
1.1 Qualitative result
negative/positive/unclear
1.2 Quantitative result
Enter value
1.3 Successful sequencing
yes/no
If 1.3 = yes
1.3.1 Virus variant
Drop-down selection
Free text
2. SARS-CoV2 antigen test
yes/no/uncertain
If2=yes
2.1 **Test system?**
2.2 **Result?**
positive, no pathogen detection, unclear

Part 2 - Study procedure

The following questions relate to the study procedure and should only be answered after the complete documentation and examination of the data.

1 The study physicians have reviewed and assessed the findings in Centraxx.
yes/no
If1=yes
1.1 Date entry
2. was the participant offered further treatment?
yes/no
3. was the participant informed of the findings?
yes/no
If 3 = no
3.1 Why not?
If 3 = yes
not reached/conspicuous findings
3.2 How was the information communicated?
in person/by telephone/fax/by email/by post

Part 1
Interview of families -children’s version
Version 4.1, 28.07.2022

Preliminary remark

In the stressful and conflict-ridden situation in an initial reception centre with people who have just fled and who may also be traumatized and whose literacy or reading skills are also uncertain, a written survey is not practical. The interview is conducted by two or more people, one of whom is a doctor, a native speaker (interpreter) and an assistant for immediate data entry via tablet.
The procedure of choice in this situation is a semi-structured interview with pre-formulated questions, most of which are open-ended. By translating and jointly classifying the answers according to subject, a valid assignment to one or more of the predefined categories is possible. In some cases, free text can also be entered into the database if there are unsuitable specifications. In many cases, multiple answers are possible.
The database is divided into different categories/blocks (see diagram, German version). The changes to the next block are marked in this guide.

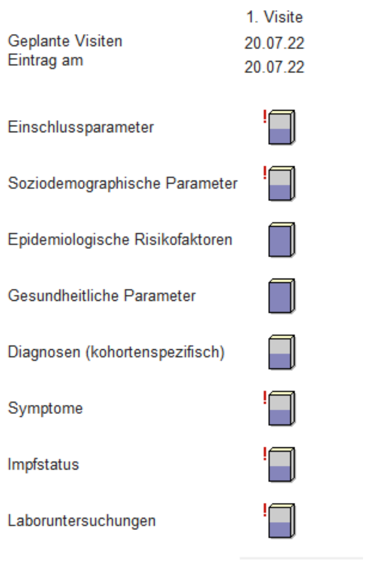

First, the data of the person signing the declaration of consent is collected and the declaration of consent is entered

Surname
Name
Date of birth
Place of birth
Address
Phone number
Email-address

ID: is assigned

The next step is to proceed to the "Inclusion parameters" category. Here you first enter some key data for each study participant

The first questions usually unfold automatically und don’t need to be asked.

1. Center
2. Cohort *(Peadiatric/Adult)*

In case of families, the “head” participant is the legal guardian. Question 3 must not be asked, but is priorily determined by the study doc.
With children, only 3.1 needs to be answered.

3. Are you the “head” participant? *(Yes/No)*
   Answers: yes/no
   If 3. = no
   3.1 What is your relationship to the “head” participant?
      Answers: Mother/father
      Brother/sister
      Grandmother/grandfather
      Daughter/son
      No familiary relationship

The next question is not put to the interviewees, but arises by itself.

4. In which language the interview was conducted?
   (German/Ukrainian/Russian/English/other)

The next question is skipped for children.

5. Who did you come to know the study?
   (Flyer/from the attending physician/friends or family/social media/other)

.

The next step is to switch to the "Social demographic parameters" category

1. Date of birth
2. Sex
   Answers: female/male/diverse/undefined
3. Country of birth
   Answers: Ukraine/another country
   3.1 If the country of birth differs from Ukraine, the country is to be selected in a drop-down menu

The next step is to switch to the "Epidemiological risk factors" category

1. Has your child had measles?
   Answers: yes/no/uncertain
2. Has your child had varicella?
   Answers: yes/no/uncertain

Now go to the "Diagnoses (peadiatric)" category

Does your child have a persistent (chronic) disease like Asthma, Diabetes or another disease?

Ask for the all the diseases using the sheet

The information on the accompanying slide is transferred by the interviewer

If no diseases are ticked on the slide, switch to "Symptoms".
Only if diseases are marked, it must be asked for drugs

Does your child have.....?
Answers: yes/no/uncertain

1. Congenital/inherited anomalies, diseases or malformations (congenital anomalies)
2. Delay of development
3. Asthma
   If 3. = yes
   3.1 Does your child take any medication? *(yes/no/uncertain)*

4. Bronchitis
   If 4. = yes
   4.1 Does your child take any medication? *(yes/no/uncertain)*

5. Diabetes mellitus
   If 5. = yes
   5.1 Does your child have insulin therapy? *(yes/no/uncertain)*

6. Epilepsy
   If 6. = yes
   6.1 Does your child take any medication? *(yes/no/uncertain)*

7. Mental distress
   If 7. = yes
   7.1 Does your child take any medication? *(yes/no/uncertain)*

8. Cancer (solid tumor)

If 8. = yes

8.1 Does your child take any medication? *(yes/no/uncertain)*

9. Haemato-oncologic diseases (leukemia)

If 9. = yes

9.1 Does your child take any medication? *(yes/no/uncertain)*

10. Immunodeficiency

10.1 Does your child take any medication? *(yes/no/uncertain)*

11. Tuberculosis

11.1 Was it a tuberculosis of the lung or at another location (pulmonary or extrapulmonary)?

Answers: pulmonary/extrapulmonary/disseminated/uncertain

1.1 When was it diagnosed for the first time?

Enter a date

1.2 Did your child get a special therapy against tuberculosis?

Answers: yes/no/uncertain

1.3 Where other drugs used than the standard therapy (Isozianid, Rifampicin, Ethambutanol, Pyrazinamid)?

Answers: yes/no/uncertain

1.4 Was a restistency against some tuberculosis-drugs constated?

Answers: yes/no/ uncertain

1.5 Was the therapy officially accomplished?

Answers: yes/no/ uncertain

1.6 Was the therapy interrupted during the flight?

Answers: yes/no/ uncertain

12. HIV/AIDS

Answers: yes/no/ uncertain

If 2 = yes

12. 1 When was the diseases diagnosed firstly?

Enter a date

12.2 Does your child take drugs against HIV?

Answers: yes/no/uncertain

Now switch to the "Symptoms" category

1 How would you describe the general status of your child's health?

Answers: excellent/very good/good/less good/bad

2 Did your child had any of mentioned symptoms in the past 3 months?

Ask for the symptoms using the sheet

The information on the accompanying slide is transferred by the interviewer

Answers: yes, no

If 2. = yes

I have/I suffer from (ask separately)

2.1 Fever?
Answers: yes/no/uncertain

2.2 Loss of appetite?
Answers: yes/no/uncertain

2.3 Sweeling of lymph nodes?
Answers: yes/no/uncertain

2.4 Headache?
Answers: yes/no/uncertain

2.5 Night sweat?
Answers: yes/no/uncertain

2.6 Loss of weight?
Answers: yes/no/uncertain

2.7 Cough?
Answers: yes/no/uncertain

2.7.1 With secretion?
Answers: yes/no/uncertain

2.7.1.1 Color of the secretion?
Answer: Clear/yellowish/yellow-greenish/brownish

2.7.1.2 Is the secretion consisting of blood?
Answers: yes/no/uncertain

2.8 Short breath (dyspnea)?
Answers: yes/no/uncertain

2.9 Gastrointestinal symptoms (e.g. diarrhea, constipation, stomachache)?
Answers: yes/no/uncertain

2.10 Neurologic symptoms (e.g. paralysis, ataxia, diplopic images)?
Answers: yes/no/uncertain

2.11 Other symptoms?

Please prepare the following questions with an explanation:

The following questions are about your own assessment of how your child deals physically and mentally with the complicated situation. The questions refer to the time here in Germany after your flight. There are no correct or wrong answers. We would like to get a better understanding of your personal experience. Then we can help you the best way. Choose the answers that fits best spontaneously.

Instruction to interviewers: If you feel that these questions are too stressful, skip questions 3 to 5

3. How would you describe your child's mental state/emotional condition at the moment? That includes your child's feelings and its ability of concentration und clear thinking.
   Answers: excellent, very good, good, less good, bad
4. Did your child feel lonely?
   Answers: never, seldom, sometimes, often, always
5. Did your child feel sad?
   Answers: never, seldom, sometimes, often, always

Now switch to the "Vaccination status" category

Note: Prepare the questions for vaccination, especially corona-vaccination with explanation

- There is no general duty for vaccination in Germany
- Even for corona there no duty, also not for adolescents
- Exception: if a child/adolescent wanst to go to kindergarten or school, vaccination against measles is required

In this study, we would like to ask for your status of vaccination. If you are not immunized, we do offer or conciliate vaccination. Vaccination remains voluntarily.

1 What is your general attitude towards vaccination?

1) I support vaccination
2) I support vaccination, but have worries concerning some vaccination
3) I am against vaccination
4) I don't have an opinion on this

If 1 = Answer 2 (worries regarding special vaccination)

1.1 I have worries concerning the following vaccination
Answers: SARS-CoV2/measles/polio/others

If 1 = Answer 1 (for vaccination)

1.2 Did your child get all the vaccination for basic immunization (according to the recommendation of MOZ)?
Answers: yes/no

2 Did you bring your child's vaccination card from Ukraine?
Answers: yes/no

3 Was your child vaccinated against one of the following diseases in Ukraine?

Ask for each vaccination using the sheet

The information on the accompanying slide is transferred by the interviewer

Answers: yes, no

Influenza in the past 6 months

Pneumococcus

Tuberculosis

Tetanus

Diphtheria

Poliomyelitis

Whooping cough (Pertussis)

Hepatitis A

Hepatitis B

Mumps

Rubella

Varicella

Meningococcus C

Haemophilus influenzae type b

Human papilloma virus (cervical carcinoma)

4 Does your child have a German vaccination card and do you have brought it with you?
Answers: yes/no

5 Was your child vaccinated against one or several of these diseases in Germany?
Answers: yes/no

Ask for each vaccination using the sheet

The information on the accompanying slide is transferred by the interviewer

Answers: yes, no

Influenza in the past 6 months

Pneumococcus

Tuberculosis

Tetanus

Diphtheria

Poliomyelitis

Whooping cough (Pertussis)

Hepatitis A

Hepatitis B

Mumps

Rubella

Varicella

Meningococcus C

Haemophilus influenzae type b

Human papilloma virus (cervical carcinoma)

6 Is your child vaccinated against SARS-CoV2?
Answers: yes/no/uncertain

6.1-4. What agent did your child get at the 1./2./3./4. Vaccination and in which country?

Ask for vaccinations and the country of vaccination using the sheet

The information on the accompanying slide is transferred by the interviewer

6.5. Why is your child not/only incompletely vaccinated against corona?

Answers:

I didn't have time for it

There were no vaccines available

I have worries concerning secondary effects

I have general doubts regarding the security of vaccines

I have general doubts regarding the efficacy of vaccines

I don't feel adequately informed on the vaccination

A doctor advised me against vaccination

Uncertain/I don't know

Only if the question for measles vaccination on sheet 14 is answered positively, other questions follow.

7 Is your child vaccinated against measles?
Answers: yes/no/uncertain
7.1 How many vaccinations has it already gotten?
7.2 Did your child get one or several vaccinations against measles in Germany?
Answers: yes/no/uncertain

This is followed by the medical examination and the change to the category "Examination findings"

1. height in cm
2. body weight in kg
3. auscultation pulmonary
normal findings/pathological
4. auscultation cor
Normal findings/pathological
6. ENT Status
Normal findings/pathological

7. vaccination scar BCG
yes/no
8. skin status
Normal findings/pathological
9. is medical treatment urgently required?
yes/no
10. injuries
Yes/no/uncertain
10.1 Localization
Head/neck
Thorax
Abdomen
Arm/hand
Leg/Foot
Multiple localizations
10.2 Type
Superficial soft tissue injury
Open wound
Bone fracture
Dislocation
Nerve injury
Vascular injury
Muscle/tendon injury
Multiple injuries

Now switch to the category **"Laboratory examination"**

1. SARS-CoV2-PCR
yes, no, uncertain
If 1 = yes
1.1 Qualitative result
negative/positive/unclear
1.2 Quantitative result
Enter value
1.3 Successful sequencing
yes/no
If 1.3 = yes
1.3.1 Virus variant
Drop-down selection
Free text
2. SARS-CoV2 antigen test
yes/no/uncertain
If2=yes
2.1 **Test system?**
2.2 **Result?**
positive, no pathogen detection, unclear

Part 2 - Study procedure

The following questions relate to the study procedure and should only be answered after the complete documentation and examination of the data.

1 The study physicians have reviewed and assessed the findings in Centraxx.
yes/no
If1=yes
1.1 Date entry
2. was the participant/family offered further treatment?
yes/no
3. was the participant/family informed of the results?
yes/no
If 3 = no
3.1 Why not?
If 3 = yes
not reached/conspicuous findings
3.2 How was the information communicated?
in person/by telephone/fax/by email/by post
